# Asymmetric dimerization of adenosine deaminase acting on RNA facilitates substrate recognition

**DOI:** 10.1093/nar/gkaa532

**Published:** 2020-06-29

**Authors:** Alexander S Thuy-Boun, Justin M Thomas, Herra L Grajo, Cody M Palumbo, SeHee Park, Luan T Nguyen, Andrew J Fisher, Peter A Beal

**Affiliations:** Department of Chemistry, University of California, Davis, CA, USA; Department of Chemistry, University of California, Davis, CA, USA; Department of Chemistry, University of California, Davis, CA, USA; Department of Chemistry, University of California, Davis, CA, USA; Department of Chemistry, University of California, Davis, CA, USA; Department of Chemistry, University of California, Davis, CA, USA; Department of Chemistry, University of California, Davis, CA, USA; Department of Molecular and Cellular Biology, University of California, Davis, CA, USA; Department of Chemistry, University of California, Davis, CA, USA

## Abstract

Adenosine deaminases acting on RNA (ADARs) are enzymes that convert adenosine to inosine in duplex RNA, a modification that exhibits a multitude of effects on RNA structure and function. Recent studies have identified ADAR1 as a potential cancer therapeutic target. ADARs are also important in the development of directed RNA editing therapeutics. A comprehensive understanding of the molecular mechanism of the ADAR reaction will advance efforts to develop ADAR inhibitors and new tools for directed RNA editing. Here we report the X-ray crystal structure of a fragment of human ADAR2 comprising its deaminase domain and double stranded RNA binding domain 2 (dsRBD2) bound to an RNA duplex as an asymmetric homodimer. We identified a highly conserved ADAR dimerization interface and validated the importance of these sequence elements on dimer formation via gel mobility shift assays and size exclusion chromatography. We also show that mutation in the dimerization interface inhibits editing in an RNA substrate-dependent manner for both ADAR1 and ADAR2.

## INTRODUCTION

RNA-editing involves altering a transcript's sequence by insertion, deletion, or modification of nucleotides resulting in a change in information content from that originally encoded in the genome (1). One of the most common forms of RNA editing in humans is the deamination of adenosine to inosine (A-to-I). Inosine functions similarly to guanosine (G) in many cellular processes such as splicing, translation, and reverse transcription by base pairing with cytidine (C) (2,3). A-to-I editing can result in the formation of alternative splice variants, alteration of microRNA processing and targeting, a change in codon sequence and suppression of activation of the innate immune system by endogenous double stranded RNAs (dsRNAs) (4–6). Dysregulation of RNA editing has been linked to neurological disorders such as epilepsy, seizures and ALS for ADAR2, and mutations within the ADAR1 gene has been linked to the autoimmune disorder Aicardi-Goutières Syndrome and Dyschromatosis Symmetrica Hereditaria (7–13). Furthermore, knock down of ADAR1 was found to be lethal to a subset of cancer cells displaying an interferon-stimulated gene signature. These studies have identified ADAR1 as a potential cancer therapeutic target (14–19). ADARs are also currently being used in directed RNA editing applications either via recruitment of endogenous ADARs with antisense guide RNAs or in engineered fusion proteins bearing ADAR deaminase domains (20–26).

**Figure 1.**
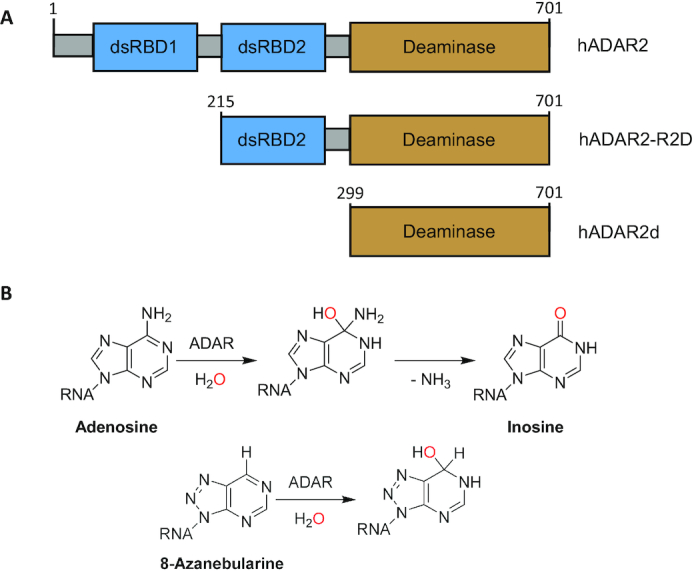
(**A**) Domain map of hADAR2 and deletion fragments. (**B**) Reaction mechanism of ADAR2 showing intermediate and 8-azanebularine (8-AN) hydrate.

A detailed understanding of the structural basis for ADAR function will advance our knowledge of the role of ADAR mutations in human disease, inform biotechnology and therapeutic applications of ADARs and accelerate the process of developing targeted inhibitors. The ADAR enzymes are modular with a C-terminal deaminase domain and double stranded RNA binding domains (dsRBDs) (Figure 1A) (27). We have previously reported the structure of human ADAR2′s deaminase domain (ADAR2d) bound to duplex RNA, which confirmed ADAR’s base-flipping mechanism, as well as shed light on protein-RNA contacts made near the edited nucleotide. However, questions remained about the role of ADAR2′s dsRBDs in substrate recognition (28). The deaminase domain of ADAR2 alone is sufficient to recognize and edit specific adenosines in certain RNA substrates, such as the glioma-associated oncogene 1 (GLI1) mRNA but is not sufficient to edit other substrates, such as the D site of the 5-HT_2C_R mRNA (29–31). The basis for this difference is not well understood. In addition, several previous studies investigated whether ADARs dimerize and the potential role of dimerization in adenosine deamination (32–34). For instance, *Drosophila* ADAR was shown to dimerize in an RNA dependent manner with the dimerization site localized to the N-terminal region of the protein. Results of FRET experiments with fluorescent fusion proteins of human ADAR1 and ADAR2 led to a similar conclusion (33,35). In contrast, disruption of RNA binding by human ADAR1 and ADAR2 with mutations at key dsRBD residues showed that ADAR2 is capable of homodimerization in an RNA independent manner and ADAR1 is able to homodimerize and complex with other RNA processing enzymes, such as Dicer, in an RNA independent manner (32,36). Thus, there is a general consensus in the literature that ADARs are able to dimerize but the location of the dimer interface and whether or not dimerization is dependent on RNA-binding is still debated (35–39). Additional structural and biochemical characterization of ADAR dimers bound to RNA would be valuable to advance our understanding of what promotes ADAR dimerization and the role dimerization plays in RNA editing.

Currently there are no structures of ADAR-RNA complexes that include both the deaminase domain and dsRBDs. An earlier study of the role of ADAR2′s dsRBDs described a highly active fragment bearing dsRBD2 and the deaminase domain (hADAR2 R2D) (40). In addition, we have shown that hADAR2 R2D efficiently edits substrates for which the deaminase domain alone is insufficient, such as the D site of the 5-HT_2C_R mRNA (31). Thus, the mechanism of RNA substrate recognition by ADAR is multifaceted and requires coordination between the deaminase domain and the dsRBDs. With that in mind, we sought to define the RNA-binding properties of ADAR2 R2D using RNA footprinting and gel shift assays along with X-ray crystallography. We then used structure-guided site-directed mutagenesis to evaluate the importance of key amino acid residues implicated in dimerization on RNA binding and adenosine deamination.

## MATERIALS AND METHODS

Unless otherwise stated, reagents were purchased from Fisher Scientific, Sigma-Aldrich, or Life Technologies. T4 polynucleotide kinase, molecular-biology-grade bovine serum albumin (BSA), and RNase inhibitor were purchased from New England BioLabs. [γ-^32^P] ATP was purchased from PerkinElmer Life Sciences. Avian myeloblastosis virus (AMV) reverse transcriptase, *Thermus filiformis* (Tfi) DNA polymerase, and dNTP mix were purchased from Promega. The QuikChange Xl II mutagenesis kit was purchased from Agilent Technologies. RNA oligonucleotides were synthesized at the University of Utah DNA/Peptide Core Facility or purchased from Dharmacon. DNA oligonucleotides were purchased from Integrated DNA Technologies. Storage phosphor imaging plates from Molecular Dynamics were imaged with a Molecular Dynamics 9400 Typhoon phosphor imager. Data were analyzed with Molecular Dynamics ImageQuant 5.2 software. Matrix Assisted Laser Desorption/Ionization (MALDI) mass spectrometry of oligonucleotide samples was performed at the Campus Mass Spectrometry Facilities, UC Davis. Oligonucleotide masses were determined with Mongo Oligo Mass Calculator 2.08.

### Expression and purification of hADAR2 double stranded RNA binding domain and deaminase domain (hADAR2-R2D) for crystallography

Protein expression and purification were carried out by modifying a previously reported protocol (41). *Saccharomyces cerevisiae* BCY123 cells were transformed with a pSc-ADAR construct encoding hADAR2-R2D E488Q (corresponding to residues 214–701). Cells were streaked on yeast minimal media minus uracil (CM-ura) plates. A single colony was used to inoculate a 15 ml CM-ura starter culture. After cultures were shaken at 300 rpm and 30°C overnight, 10 ml of starter culture was used to inoculate each liter of yeast growth medium. After cells reached an OD_600_ of 1.5 (∼20–24 h) cells were induced with 110 ml of sterile 30% galactose per liter and protein was expressed for 6 h. Cells were collected by centrifugation at 5000×g for 10 min and stored at -80°C. Cells were lysed in 750 mM NaCl in buffer A (20 mM Tris–HCl, pH 8.0, 5% glycerol, 35 mM imidazole, 1 mM BME and 0.01% Triton X-100) with a microfluidizer. Cell lysate was clarified by centrifugation (39 000×g for 25 min). Lysate was passed over a 5 ml Ni-NTA column equilibrated with buffer A with 750 mM NaCl, which was then washed in three steps with 50 ml of lysis buffer, wash I buffer (buffer A + 300 mM NaCl), and wash II buffer (buffer A + 100 mM NaCl). Protein was eluted with a 35–300 mM imidazole gradient in wash II buffer over 80 min at a flow rate of 1 ml/min. Fractions containing target protein were pooled and further purified on a 2 ml GE Healthcare Lifesciences Hi-Trap Heparin HP column in wash II buffer without BME. The His_10_ fusion protein was washed with 50 ml of wash II buffer without BME and eluted with a 100–1000 mM NaCl gradient over 60 min at a flow rate of 0.8 ml/min. Fractions containing target protein were pooled and cleaved with an optimized ratio of 1 mg of TEV protease per 1 mg of protein. Cleavage was carried out for 2 h before the product was passed over another Ni-NTA column with a flow rate of 0.5 ml/min. The flow-through and wash were collected, dialyzed against 20 mM Tris, pH 8.0, 200 mM NaCl, 5% glycerol and 1 mM BME, followed by concentration to just under 1 ml for gel filtration on a GE Healthcare HiLoad 16/600 Superdex 200 PG column. Fractions containing purified protein were pooled and concentrated to 7–9 mg/ml for crystallization trials.

### Purification of 8-AN containing RNAs

The 8-azanebularine (8-AN) phosphoramidite was synthesized as previously described (42). The 8-AN phosphoramidite was provided to the DNA/Peptide Core Facility at the University of Utah to be incorporated in RNA strands as previously described (43). Single-stranded RNAs were purified by denaturing PAGE and visualized with UV shadowing. Bands were excised from the gel, crushed, and soaked overnight at 4°C in 0.5 M NH_4_OAc, 0.1 mM EDTA. Polyacrylamide fragments were removed with a 0.2 μm filter, and the RNA was ethanol precipitated followed by 70% ethanol wash. Desalting of the RNA was accomplished by three rounds of concentration to under 100 μl and subsequent addition of 300 μl nuclease-free water in a 3000 MWCO Amicon Ultra 0.5 ml centrifugal filter (Millipore Sigma). After concentration, the RNA solutions were lyophilized to dryness, resuspended in nuclease-free water, quantified by UV absorbance at 260 nm, and stored at –70°C. Oligonucleotide mass was confirmed by matrix-assisted laser desorption ionization mass spectrometry using 3-hydroxypicolinic acid as the matrix. Unmodified RNA strands were purchased from Horizon Dharmacon and purified as described above. Duplex RNA was hybridized in a 1:1 ratio by heating to 95°C for 5 min and slow cooling to 30°C.

### Crystallization of the hADAR2–R2D E488Q–RNA complex

Crystals of the hADAR2–R2D E488Q–GLI1 RNA complex were grown at room temperature by the hanging-drop vapor-diffusion method. Using a TTP-Labtech mosquito, a 200 nl solution containing 5.5 mg/ml protein and 50 μM of GLI1 32 bp RNA (1:0.5 ADAR/RNA molar ratio) was mixed with 200 nl of 50 mM MOPS, pH 7.0, 20% (w/v) PEG 4000, and 100 mM NaCl. Crystals took approximately a month to grow. A cluster of crystals was broken apart and a single cuboid-shaped crystal ∼100 μm in size was soaked briefly in a solution of mother liquor plus 30% ethylene glycol before flash cooling in liquid nitrogen. Data were collected with 1.0° oscillations on beamline 12–2 at the Stanford Synchrotron Radiation Lightsource. Crystals display anisotropic X-ray diffraction with some diffraction extending beyond 2.2 Å resolution. However, the resolution was isotropically truncated to 2.8 Å resolution to generate a robust complete data set.

### Crystallographic data processing, structure solution and refinement

X-ray diffraction data for the ADAR2-R2D E488Q GLI1-bound structure were processed with XDS and scaled with XSCALE (44). The structure was determined by molecular replacement using PHENIX (45). The hADAR2d GLI1-bound crystal structure (PDBID: 5ED2) was used as the deaminase domain and dsRNA models together with an ensemble of dsRBD structures (PDBIDs: 2L3J, 2B7V, 5DV7 and 2L2K). The program identified the placement of two deaminase domains, one RNA duplex, and a single dsRBD. The structure was refined with PHENIX including NCS and zinc coordination restraints (46). Ideal zinc-ligand distances were determined with average distances found for similar coordination models in the PDB database, including deposited ADAR2 structures. Table 1 shows statistics in data processing and model refinement. The asymmetric unit includes two protein molecules complexed with RNA. The whole of the double stranded RNA binding domain (residues 215–318) of monomer A, as well as the C-terminal residues 700 and 701 were disordered and were therefore not included in the model. The first 20 residues (215–233), 5′ binding loop residues 462–475 and C-terminal proline (701) of monomer B were disordered and were therefore not included in the model.

**Table 1.** Data processing and refinement statistics for ADAR2-R2D E488Q:GLI1 8-AN 32mer PDB: 6VFF

Synchrotron (beamline)	SSRL (12–2)
Wavelength (Å)	0.97946
Space group	*C*2
Unit cell parameters (Å)	*a* = 170.0, *b* = 63.21, *c* = 142.20
Resolution range (Å)	37.48–2.80 (2.87–2.80)
No. observed reflections	178 826 (12 253)
No. unique reflections	32 384 (2260)
Completeness (%)	97.3 (92.1)
I/σ (I)	14.14 (1.56)
*R*_merge_^a^ (%)	9.4 (118.1)
CC_1/2_	99.8 (61.1)
**Refinement statistics**	
*R*_factor_^b^ (%)	0.1965
*R*_free_^b^ (%)	0.2483
RMS bond length (Å)	0.008
RMS bond angle (°)	1.247
**Ramachandran plot statistics** ^c^	
Favored (%)	93.09
Allowed (%)	5.06
Outliers (%)	1.85
**No. of atoms**	
Protein	6403
RNA	1357
Inositol hexakisphosphate (IHP)	72
Zn	2
Waters	36
***B* factors**	
Protein	78.36
RNA	97.23
IHP	63.44
Zn	56.79
Water	62.10

^a^ *R*_merge_ = [∑_h_∑_i_|*I*_h_ – *I*_hi_|/∑_h_∑_i_*I*_hi_] where *I*_h_ is the mean of *I*_hi_ observations of reflection h. Numbers in parenthesis represent highest resolution shell.

^b^ *R*-factor and ^c^*R*_free_ = ∑||*F*_obs_| – |*F*_calc_|| / ∑|*F*_obs_| x 100 for 95% of recorded data (*R*-factor) or 5% data (*R*_free_).

^c^Ramachandran plot statistics from MolProbity (73).

### Expression and purification of hADAR2-R2D for *in vitro* assays

Histidine-tagged human ADAR2-R2D mutant proteins were expressed in *S. cerevisiae* strain BCY123 and purified as described above with the following modifications. Cell lysate was purified by a 0.22 μm filter after centrifugation and loaded three times through a 5 ml Ni-NTA Superflow column (Qiagen) at 2.5 ml/min. The column was washed with 100 ml of buffer A with 750 mM NaCl, followed by 50 ml washes of wash I and wash II 2.5 ml/min. Elution was performed by a 45 ml gradient from wash II to elution buffer (wash II with 300 mM imidazole). Fractions were analyzed by SDS-PAGE and selected fractions were pooled and loaded at 1.5 ml/min on a 5 ml HiTrap Heparin HP column from GE. The column was washed with 30 ml of heparin 1 buffer at 1.5 ml/min and eluted with a 45 ml gradient from wash II buffer without BME to wash II buffer with 1000 mM NaCl without BME. Fractions were analyzed by SDS-PAGE and selected fractions were pooled and concentrated to <300 μl in a 30 000 MWCO Amicon Ultra 4 centrifugal filter at 5000 × g and 4°C. TEV protease cleavage and gel filtration steps were omitted. Buffer exchange was accomplished via three rounds of concentration to <300 μl and subsequent addition of 3 ml of storage buffer (buffer II with 20% glycerol). After final centrifugation, protein concentrations were determined with BSA standards, as visualized by SYPRO Orange staining on SDS-PAGE gels. The purified proteins were stored at –70°C.

### Site-directed mutagenesis

Mutagenesis of the hADAR2 R2D and full-length constructs was carried out via PCR site-directed mutagenesis using QuikChange XL Site-Directed Mutagenesis Kit (Agilent) with the primers listed in the sequence table. All primers were purchased from IDT and were PAGE purified as described above but were desalted by ethanol precipitation and 70% ethanol wash instead of filter centrifugation. Sequences for mutant plasmids were confirmed by Sanger sequencing. See Supplementary Table S1. for primer sequences.

### Preparation of ^32^P labeled 91bp 8-AN hGLI1 RNA for MPE-Fe (II) footprinting

Oligonucleotides were purified by denaturing PAGE and extracted by the crush and soak method similarly to RNA used for crystallography but were desalted only by ethanol precipitation and 70% ethanol wash. The hGLI1 91 5′end (80 pmol) was radiolabeled with [γ-^32^P] at the 5′ end with T4 PNK as described previously (47). ^32^P labeled GLI1 91 5′ end (33 mer) RNA was phenol-chloroform extracted, ethanol precipitated, desalted with 70% ethanol, dried, resuspended in nuclease free water and hybridized 1:1:1:1 with 5′-phosphorylated hGLI1 3′ Extended top (28mer), 5′-phosphorylated hGli1 91 3′end (30mer) and 90 base complementary DNA splint (GLI1 DNA splint) in 1× T4 RNA ligase 2 buffer by heating to 95°C and slowly cooled to room temperature. T4 RNA ligase 2 (5 μl of 10 U/μl) was added along with 3 μl of 1.6 U/μl RNase inhibitor to a final reaction volume of 140 μl. The sample was incubated at room temperature overnight. Ligated product was phenol:chloroform extracted, ethanol precipitated and dried. Ligated product was resuspended in nuclease free water and DNA was digested with RQ1 RNase free DNase-I in 1× DNase buffer for one hour at 37°C. The splint ligation products were PAGE purified as described above. Labeled RNAs were hybridized with the complementary GLI1 bottom strand RNA in 10 mM Tris–HCl pH 7.5 and 100 mM NaCl. See Supplementary Table S2. for oligonucleotide sequences.

### *In vitro* transcription of hGLI1 91 bottom strand RNA for footprinting

The complementary strand to the 91 base 8-AN was transcribed and from a linearized plasmid template using the ThermoFisher Megascript T7 kit (AM1334). Transcription reaction was incubated at 37°C overnight. Product was purified by electrophoresis on 8% denaturing PAGE gel, crushed and soaked overnight at 4°C in 0.5 M NH_4_OAc, and 0.1 mM EDTA. Polyacrylamide fragments were removed using a 0.2 μm filter followed by ethanol precipitation. The RNA was dried on a speed-vac and stored at −20°C.

### MPE-Fe (II) cleavage footprinting assay

Footprinting reactions were carried out in 20 μl volumes. 5′ ^32^P-labeled 8-AN GLI1 91 bp duplex RNA (500 fmoles) and 20 ng of yeast tRNA was mixed with 40 nM hADAR2-R2D-E488Q, 200 nM hADAR2d-E488Q, or no enzyme in a simplified ADAR binding buffer and incubated for 10 min at 30°C. A premixed solution of methidiumpropyl-EDTA (MPE) (Sigma M6164) and freshly dissolved ammonium iron(II) sulfate was added to a final concentration of 1 μM MPE and 2 μM Fe(II) and incubated for 3 min at 30°C. The cleavage reaction was started with the addition of 20 mM freshly dissolved sodium ascorbate. Cleavage reactions were incubated for 10 min at 30°C and quenched by addition of 180 μl of 1% glycerol. Final footprinting conditions were 60 mM KCl, 10 mM NaCl, 7.5 mM Tris pH 7.5, 0.1 mM betamercaptoethanol, 1 μM MPE, 2 μM ammonium iron(II) sulfate, 20 mM sodium ascorbate. Samples were phenol:chloroform extracted, ethanol precipitated, washed with 70% ice cold ethanol and dried. Samples were resuspended in 10 μl formamide loading buffer (80% w/v formamide, 2 mM EDTA pH 8.0), heated to 95°C for 7 min and run on a 12% denaturing PAGE sequencing gel for 4.5 h at 20 W. Gel was dried and exposed on a phosphor-image screen for 18 h. Phosphor screen was imaged on a GE typhoon and cleavage patterns analyzed by linear densitometry using ImageQuant 5.2. One sample was included which was processed identically to the 0 nM enzyme sample except sodium ascorbate was omitted. Nuclease T1 and alkaline hydrolysis sequencing was carried out as described in Ambion catalog 2283. T1:2 picomoles of single stranded 5′ ^32^P labeled 8-AN GLI1 91 was mixed with 1 μl 1 mg/ml yeast tRNA and 17 μl supplied RNA sequencing buffer. Sample was heated at 50°C for 5 min and returned to room temperature. T1 nuclease (3 μl) diluted to 0.1 U/μl in 1× T1 reaction buffer was added and reaction was incubated 15 min at room temperature. The provided inactivation/precipitation buffer was added (60 μl), the sample was vortexed and incubated at –80°C for 30 min. Sample was centrifuged at 4°C for 15 min to pellet and the supernatant discarded. The pellet was washed with 70% ethanol, dried and resuspended in 15 μl formamide loading buffer, heated to 95°C for 7 min. The resulting solution (5 μl) was loaded alongside MPE footprinted samples. Alkaline hydrolysis: 1.2 pmol single stranded 5′ ^32^P labeled 8-AN GLI1 91 was mixed with 1 μl 1 mg/ml yeast tRNA and 8 μl supplied alkaline hydrolysis buffer (final volume 11 μl). Sample was heated to 95°C for 2 min and placed on ice. Sample was mixed 1:1 with formamide loading buffer, heated to 95°C for 7 min and 8 μl was loaded alongside MPE footprinting samples. See Supplementary Figure S1 for linear plot of optical density of MPE-Fe cleavage fragments.

### Gel mobility shift assay of duplex RNA and ADAR

Oligonucleotides were 5′-end labeled using [γ-^32^P] ATP (6000 Ci/mmol) and T4 polynucleotide kinase, purified by 19% denaturing polyacrylamide gel in 1× TBE, visualized by storage phosphor autoradiography, excised, and extracted by crush and soak. The labeled oligonucleotide was hybridized to its unlabeled complement by heating to 95°C for 5 min in 1× TE and 200 mM NaCl, followed by slow cooling to room temperature. Samples containing less than 1.1 nM RNA and different concentrations of hADAR2-R2D mutants (64, 32, 16, 8, 4, 2, 1, 0.5, 0.25 and 0 nM) were equilibrated in 20 mM Tris–HCl, pH 7.0, 3.5% glycerol, 0.5% DTT, 60 mM KCl, 20 mM NaCl, 0.1 mM BME, 1.5 mM EDTA, 0.003% NP-40, 160 units/ml RNasin, 100 μg/ml BSA, and 1.0 μg/ml yeast tRNA for 30 min at 30°C. Samples were loaded onto a running 6% nondenaturing polyacrylamide gel (79:1 acrylamide:bisacrylamide) and electrophoresed in 1× TBE buffer at 4°C for 90 min. The gels were dried on a gel drier (Biorad) for 60 min at 80°C under vacuum followed by exposure to storage phosphor imaging plates (Kodak) for 3 h in the dark. After exposure, the dried polyacrylamide gels were removed, and the phosphor imaging plates were scanned by Typhoon Trio Variable Mode Imager (GE Healthcare). See Supplementary Table S2 for RNA sequences.

### Analytical size exclusion chromatography of ADAR2-R2D and duplex RNA

For analytical gel filtration analysis, 25 μl of 10 μM ADAR2-R2D, with or without 5 μM hGLI1 3′ extend RNA, was injected onto a Superdex increase 5/150 GL gel filtration column using an AKTA FPLC system (GE healthcare). The protein was eluted with 20 mM Tris–HCl pH 8.0, 200 mM NaCl, 5% glycerol and 1 mM BME. The elution profile was analyzed by the evaluation tools included in the Unicorn 7.4 software package. See Supplementary Figure S2 for calibration curve generated for gel filtration column using molecular weight standards (Biorad and Sigma).

### Preparation of *in vitro* transcribed 5HT2c-RNA for *in vitro* deamination kinetics

The 5HT2c-R transcript was prepared as described previously (30). In brief, a plasmid containing a 332 nt truncation of the 5HT2c R mRNA was inserted within the SmaI site of a pGE plasmid. The plasmid was linearized with BamHI and the RNA was transcribed and purified as above. See Supplementary Figure S3. for transcribed RNA sequence.

### Deamination kinetics with ADAR2-R2D and 5HT_2C_-R

Deamination reactions had a final volume of 10 μl with concentrations of 10 nM RNA and 100 nM ADAR2-R2D. The final reaction solution contained 17 mM Tris pH 7.4, 60 mM KCl, 16 mM NaCl, 2 mM EDTA, 0.003% Nonidet *P*-40, 0.5 mM DTT, 1.0 μg/ml yeast tRNA, 160 units/ml RNasin. The reaction was incubated at 30°C. Time points include 0, 0.5, 1, 3, 5, 10, 15, 30 and 60 min. The zero point consisted of omitting protein, and immediate quench. Reactions were quenched by addition of 190 μl of 95°C nuclease free H_2_O and incubation for 5 min at 95°C. cDNA was generated from RNA via RT-PCR using Access RT-PCR kit (Promega) for 24 cycles. PCR product was purified using DNA Clean and Concentrator kit (Zymo). Purified samples were subjected to Sanger Sequencing and sequence traces were analyzed by 4Peaks (Nucleobytes) to quantify percent editing. Rate constants were calculated by fitting the time course to the equation: [*P*]_*t*_ = α[1 = e^−*k*obs*t*^] where [*P*]_*t*_ is percent edited, α is the end point fitted to 95% and *k*_obs_ is the observed rate constant. See Supplementary Table S1. for cDNA sequencing primers.

### Cellular editing of endogenous substrates in HEK293T cells

HEK293T cells were cultured in Dulbecco's modified Eagle's medium (DMEM), 10% fetal bovine serum (FBS) and 1% anti–anti at 37°C and 5% CO_2_. At 70–90% confluency, 8 × 10^4^ cells for ADAR2 experiments, or 6.4 × 10^3^ cells for ADAR1 experiments were seeded into a 96-well plate. After 24 h, cells were transfected with 750 ng pcDNA3.1 plasmid containing ADAR1 p110 or full length hADAR2 WT or mutant with HA tag. Transfection was carried out using Lipofectamine 2000. After incubation of transfection reagent and plasmid in Opti-MEM, the solution was added to designated well and incubated at 37°C and 5% CO_2_ for 48 h. Total RNA was isolated using RNAqueous Total RNA Isolation Kit and DNase treated with RQ1 RNase-free DNase (Promega). Nested PCR was performed by first using Access RT-PCR Kit (Promega) for 20 cycles for ADAR1 or 24 cycles for ADAR2 followed by a second PCR using Phusion Hot Start DNA Polymerase for 30 cycles for ADAR1 or 19 cycles for ADAR2. The PCR products were purified by agarose gel and Qiagen Gel Extraction Kit. The purified product was subjected to Sanger sequencing and sequence traces were analyzed by 4Peaks (Nucleobytes) to measure percent editing. See Supplementary Table S1 for cDNA sequencing primers.

## RESULTS

### Footprinting of a trapped ADAR2–R2D–RNA complex indicates dsRBD2 binds 3′ to an editing site

**Figure 2.**
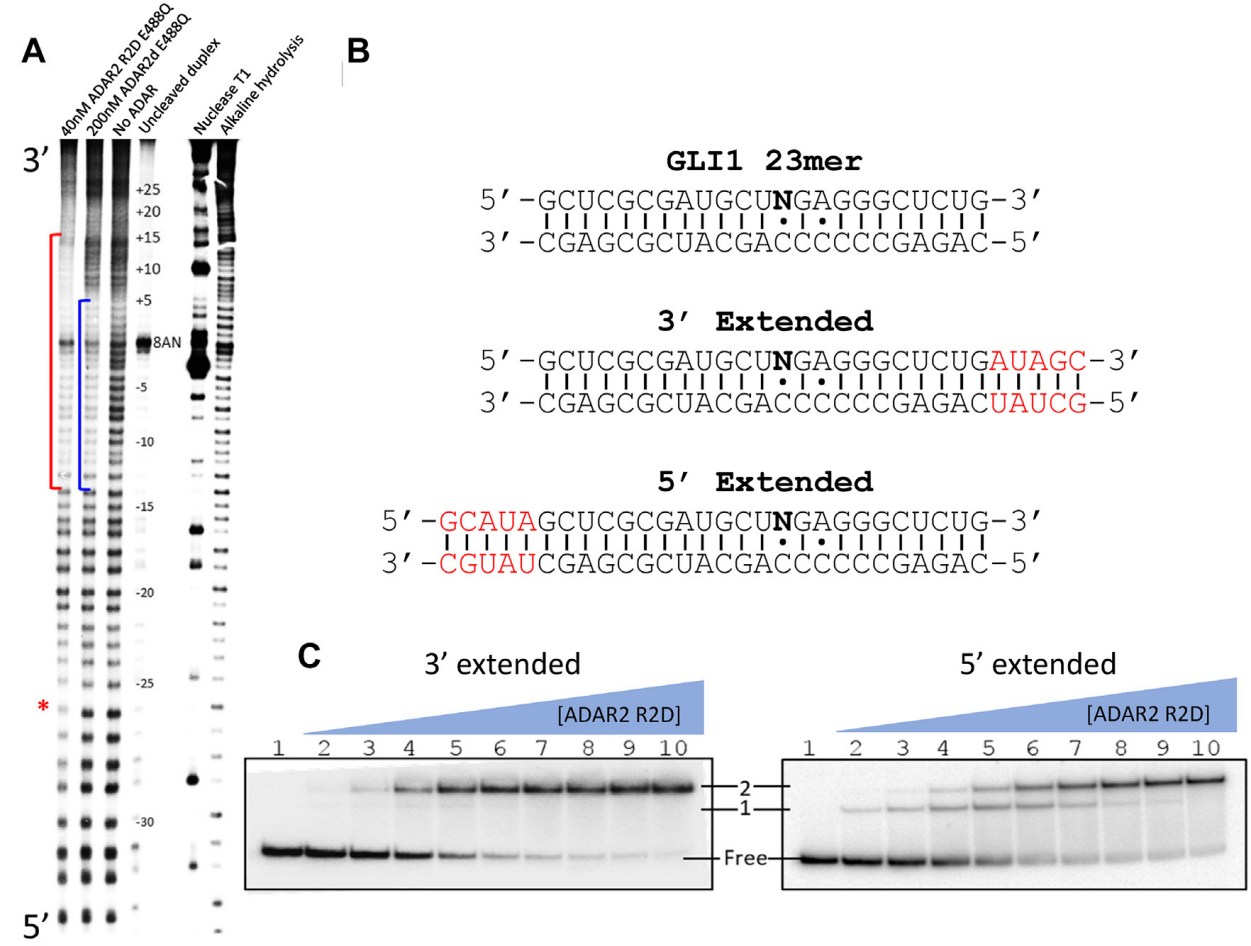
MPE•Fe footprinting and electrophoretic mobility shift assay (EMSA) of 8-AN containing RNA. (**A**) Polyacrylamide gel of 5′ ^32^P labeled RNA fragments resulting from limited MPE•Fe cleavage. Red brackets represent regions in lane 1 that show strong cleavage protection by hADAR2-R2D, the blue brackets represent regions in lane 2 that show strong cleavage protection by hADAR2d. Red asterisk indicates site of protection by ADAR2-R2D 5′ to editing site. (**B**) Sequence of GLI1 RNA used for gel shift assay. 8-AN is represented as N. Extensions from 23 bp duplex ligand of hADAR2d are shown in red. (**C**) Gel shift of hADAR2-R2D with 3′ extended or 5′ extended RNA duplex. Protein concentrations are as follows: lane 1: no protein, lanes 2–10: 0.25, 0.5, 1, 2, 4, 8, 16, 32, 64 nM.

We began our investigation by searching for differences in protein-RNA binding between ADAR2d, and ADAR2-R2D with the goal of identifying candidate RNAs for X-ray crystallography. Initially, we sought to compare the size of the binding sites for ADAR2d and ADAR2 R2D on a long duplex RNA using footprinting. The effect of substituting an RNA substrate's target adenosine with the modified nucleoside 8-azanebularine (8-AN) has been well established (48). Here, we took advantage of the property of 8-AN to mimic ADAR’s reactive tetrahedral intermediate, in order to form a high affinity protein–RNA complex near the center of a long duplex (Figure 1B). We first generated an 8-AN bearing 91 bp RNA duplex labeled at the 5′ end of one strand with ^32^P. To enhance protein–RNA complex formation, we also used the hyperactive ADAR2 mutation E488Q, which exhibits a higher binding affinity to duplex RNA (49). We then compared the binding sites for ADAR2d E488Q and ADAR2 R2D E488Q using footprinting with the nonspecific duplex RNA cleavage reagent methidium-propyl EDTA Fe (II) (50). Comparing the footprint of ADAR2d E488Q to the unbound RNA revealed sites of protection around the 8-AN that align well with our previously reported structures (Figure 2A). The ADAR2-R2D E488Q’s footprint extended approximately 10 bp past the ADAR2d E488Q footprint in the 3′ direction relative to the 8-AN position. These results are consistent with the previously reported NMR structure in which the rat homologue of ADAR2′s dsRBD2 appears to interact along a 10 bp binding site (51) and suggest ADAR2-R2D’s dsRBD2 binds on the 3′ side of the editing site on this RNA. It should be noted that we also observed a region of weak protection by ADAR2-R2D E488Q approximately 25–32 bp in the 5′ direction relative to 8-AN, which is not observed for hADAR2d.

### Gel shift pattern displayed by ADAR2-R2D and an 8-AN-containing RNA duplex suggests formation of a stable complex involving an ADAR dimer

Electrophoretic mobility gel shift assays (EMSA) are useful for the study of protein-RNA interactions in solution (52). In previous work, we used EMSA and X-ray crystallography to characterize the complex formed between ADAR2d and a 23 bp duplex whose sequence is derived from the GLI1 mRNA (28). To generate an RNA that would accommodate the binding site of ADAR2-R2D defined in the footprinting experiments above, we extended that 23 bp duplex by 5 bp in the 3′ direction from the 8-AN position (Figure 2B). For comparison, we also generated 28 bp RNA duplex by extending the duplex in the 5′ direction (Figure 2B). We predicted that the RNA extended in the 3′ direction may bind differently than the RNA extended in the 5′ direction given the directionality of binding revealed in the footprint observed with ADAR2-R2D E488Q (Figure 2). Indeed, comparison of the two gel shifts reveals two different RNA bound species. The gel containing RNA extended 5′ shows a singly-shifted band at low protein concentrations that transitions to a super shift at high protein concentrations while the 3′ extended RNA shows almost exclusively a super shift even at low protein concentrations (Figure 2C). In fact, the banding pattern of the 5′ extended RNA appears to be similar to that observed with the original 23 bp RNA duplex (Supplementary Figure S4a). This gel shift pattern suggests formation of a high affinity 2:1 protein-RNA complex is promoted when sufficient duplex is present 3′ of the editing site.

### ADAR2-R2D + RNA crystal structure reveals an asymmetric protein dimer bound to RNA

**Figure 3.**
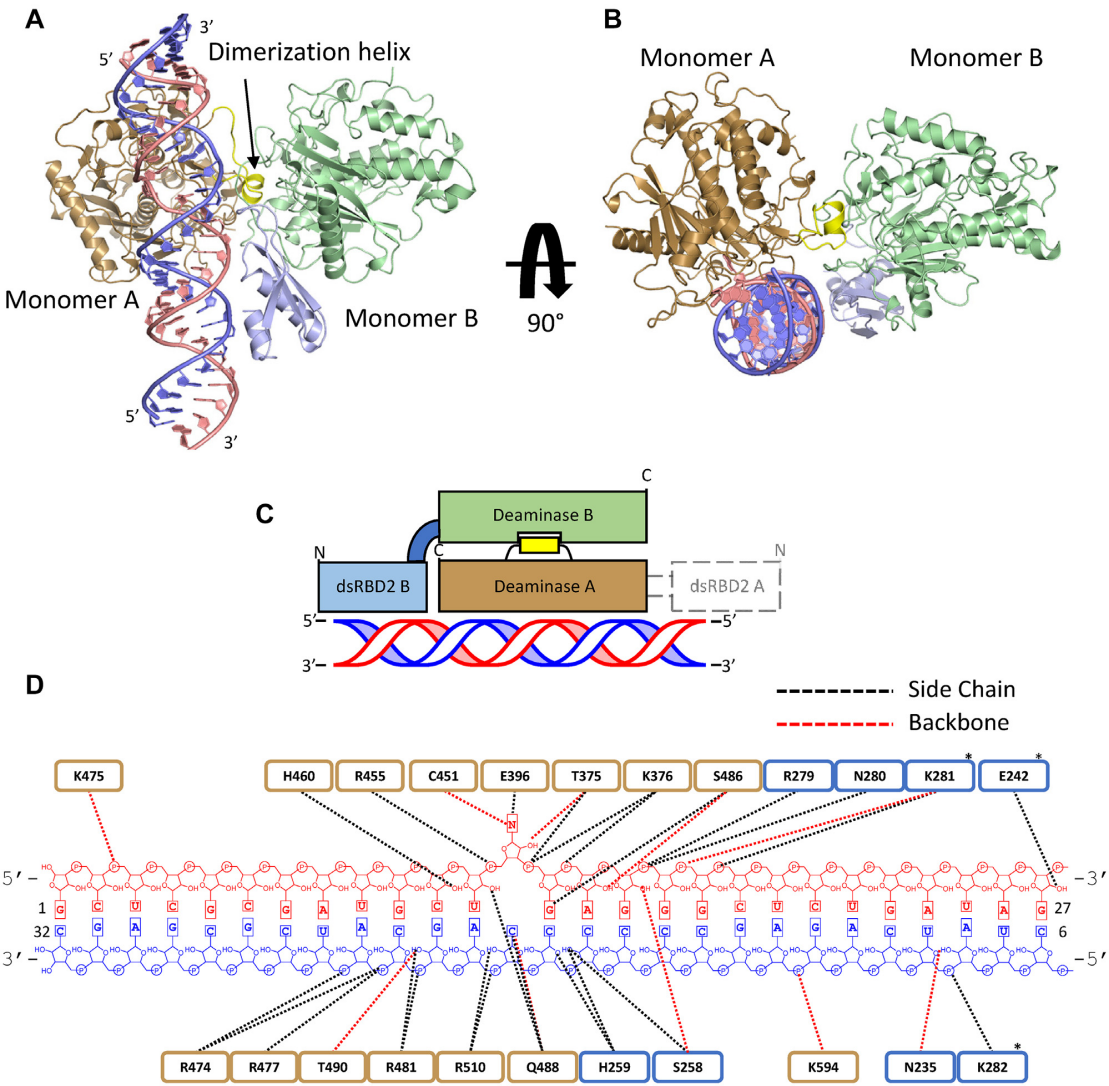
Structure of hADAR2-R2D E488Q bound to the GLI1 32 bp RNA at 2.8 Å resolution. (**A**) View of the structure perpendicular to the dsRNA helical axis. (**B**) View of the structure along the dsRNA helical axis. (**C**) Cartoon schematic of the asymmetric protein dimer. Colors for ADAR2 domains and RNA strands correspond to those used in A and B. Electron density for dsRBD2 on monomer A was not resolved. (**D**) Summary of contacts between hADAR2-R2D E488Q and GLI1 32 bp RNA duplex. Brown represent RNA contacts to deaminase A; blue represents RNA contacts to dsRBD2 of monomer B. Asterisk represent potential contacts that lack full side-chain electron density.

**Figure 4.**
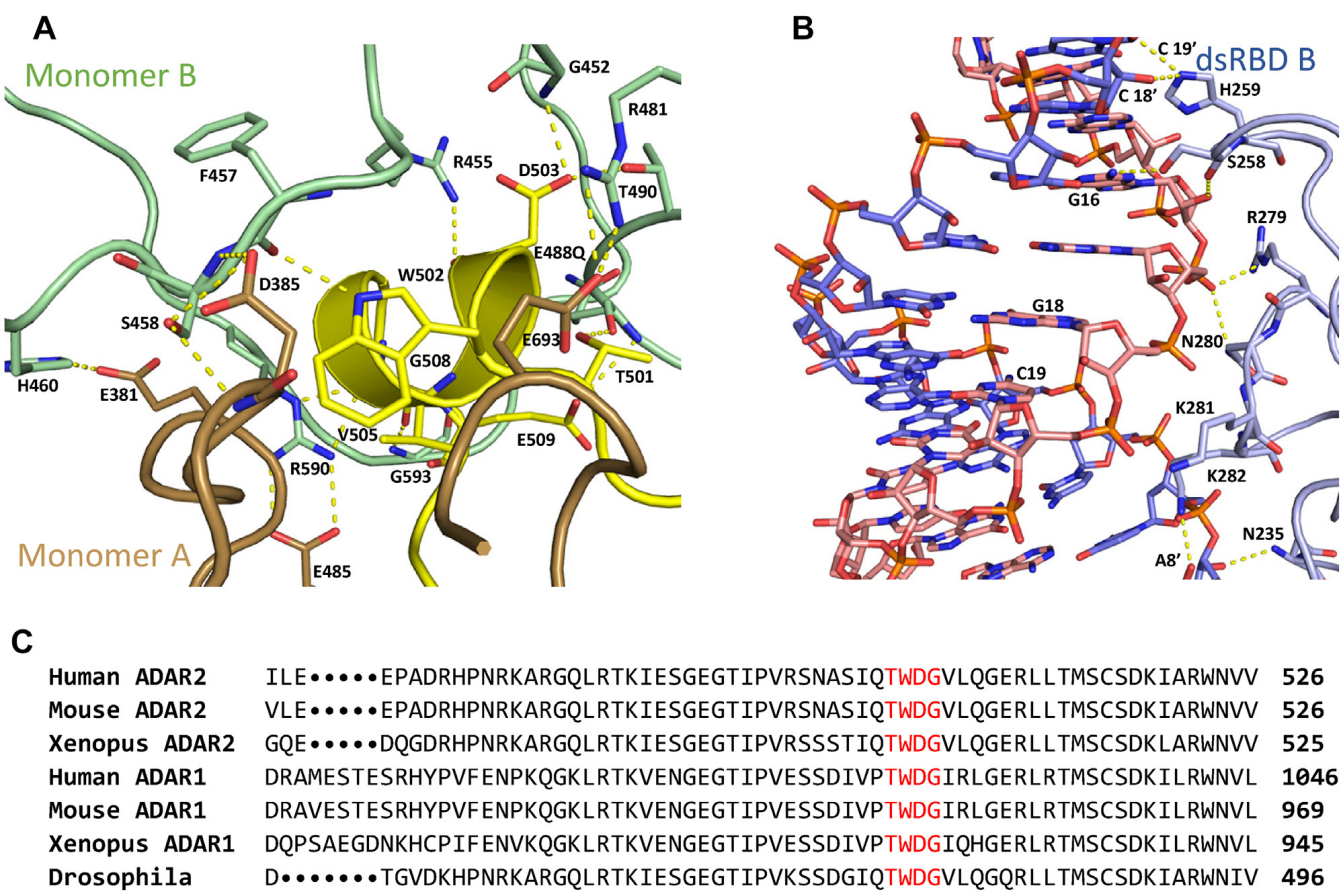
ADAR contacts observed in protein dimer. (**A**) Protein–protein contacts within dimerization interface. Monomer A is primarily colored brown, the dimerization helix of Monomer A is colored yellow, Monomer B is colored green. (**B**) Contacts between dsRBD2 B and RNA duplex. dsRBD B is labeled in light blue, 8-AN containing RNA strand is labeled in light pink, the complementary strand is labeled in blue. (**C**) Representative sequence alignment for ADARs showing sequence conservation in dimerization helix. Conserved residues contained within ADAR2′s dimerization helix are highlighted red.

We next sought to obtain a high-resolution crystal structure of the ADAR2 R2D + dsRNA complex. We were able to form protein/RNA crystals using a 32 bp 8-AN-containing duplex and ADAR2-R2D E488Q diffracting X-rays beyond 2.8 Å resolution (Table 1) (Figure 3). The RNA-bound protein presents itself as an asymmetric dimer. The deaminase domain of one ADAR2-R2D monomer is involved in direct RNA binding to the flipped-out 8-AN base, comparable to our previously reported ADAR2d/RNA structures (28). The second ADAR2-R2D molecule directly interacts with the RNA-bound deaminase via an extensive network of protein-protein contacts. We refer to these molecules as monomers A and B, respectively (Figure 3A–D). The deaminase domain of monomer A bound to RNA aligns well to our previously reported structures, (RMSD 0.43 Å to PDBID: 5ed2, for 2898 equivalent atoms of monomer A and RNA). The deaminase domain of monomer B uses a significant portion of the surface previously shown to bind RNA, including its catalytic pocket, to contact a short α-helix from the monomer A deaminase domain. The deaminase domain of monomer B does not directly engage the RNA duplex, instead the dsRBD2 of monomer B contacts the RNA. Indeed, dsRBD2 B binds the RNA duplex on the 3′ side of the 8-AN, as predicted from the footprinting data (Figure 4A, B). Several dsRBD residues such as N280, K281, E242 and K282 make phosphodiester backbone or 2′-OH contacts as described earlier for other dsRBD–RNA structures and for the RNA-bound rat ADAR2 dsRBD structures (51,53–55). While dsRBD2 B interacts with the RNA, no clear electron density presented itself for the dsRBD2 of monomer A. Within the structure, the linker bridging deaminase A to its dsRBD is directed in the 5′ direction relative to the 8-AN bearing strand of the RNA duplex, a void within the unit cell lacks electron density to model the dsRBD. It is most likely that the disordered dsRBD2 A resides in this location.

The buried surface area between the two protein monomers is large (1043 Å^2^ for each monomer) with low temperature factors, suggesting the interface is quite stable. Interestingly, residues such as G452, R455, F457, S458, H460, R481, Q488, T490, R590 and G593 in monomer B, which comprises the catalytic pocket or RNA binding surface, instead participate in forming protein-protein contacts to an exposed α-helix from monomer A (consisting of residues 501–509), which we will refer to here as the dimerization helix (Figure 4A). Key residues within the dimerization helix of monomer A are in close contact with what would typically be the RNA binding surface of monomer B, for example, the side chains of T501 and W502 of monomer A make backbone interactions with residues Q488 and F457, respectively, from monomer B. The side chain of E509 also interacts with the main chain of Q488 of monomer B. In another contact, the carbonyl oxygen of G508 in the dimerization helix hydrogen bonds with the main-chain amide nitrogen of G593 of monomer B. It is interesting to note that this highly conserved G593 makes a close contact to the phosphodiester backbone of the RNA substrate in the A monomer and our previous ADAR2d-RNA structure and mutating this to alanine greatly diminishes ADAR2d activity (28). Additionally, H460 of monomer B appears to interact with the side chain of E381 of monomer A in contrast to H460 of monomer A which is involved in forming an interaction with a 2′ OH in the RNA duplex.

D503 of the dimerization helix makes key protein-protein interactions. The side chain of D503 is in hydrogen bonding distance to main chain of G452 and the side chain of T490. Residue T490 is of interest due to previous studies identifying the mutation T490A in the deaminase domain of ADAR2 as particularly deleterious to base flipping and deamination (49). Although T490A was proposed to negatively affect the base flipping step of ADAR2 by disrupting the flipping loop conformation, it is possible that it also leads to lower activity by blocking an interaction with D503 in the dimerization helix.

Three arginine residues of the B monomer make significant interactions with the A monomer. Specifically, R481 and R590 ion-pair with side chains of E693 and E485 respectively of monomer A (Figure 4a). Additionally, R590 hydrogen-bonds to main chain of V505 of the dimerization helix of monomer A. R455 of monomer B also hydrogen bonds to the dimerization helix of monomer A, where it interacts with main chain of D503. These three arginine residues, which encircle the catalytic pocket of monomer B, mediate substrate RNA interactions in the A monomer and in our previous ADAR2d-dsRNA structures (28). However, this new structure shows these arginines also play an important role in mediating protein-protein interactions in the asymmetric dimer.

Multiple species sequence alignment of ADAR proteins shows a high degree of conservation for residues present at the protein-protein interface. Conserved residues found on the dimerization helix (i.e. ^501^TWD^503^) were not previously known to have a role in editing despite their high degree of conservation (Figure 4c). The structure presented here suggested these conserved residues play an important role in mediating protein dimerization.

### Mutation of the dimer interface disrupts dimer formation and RNA editing

**Figure 5.**
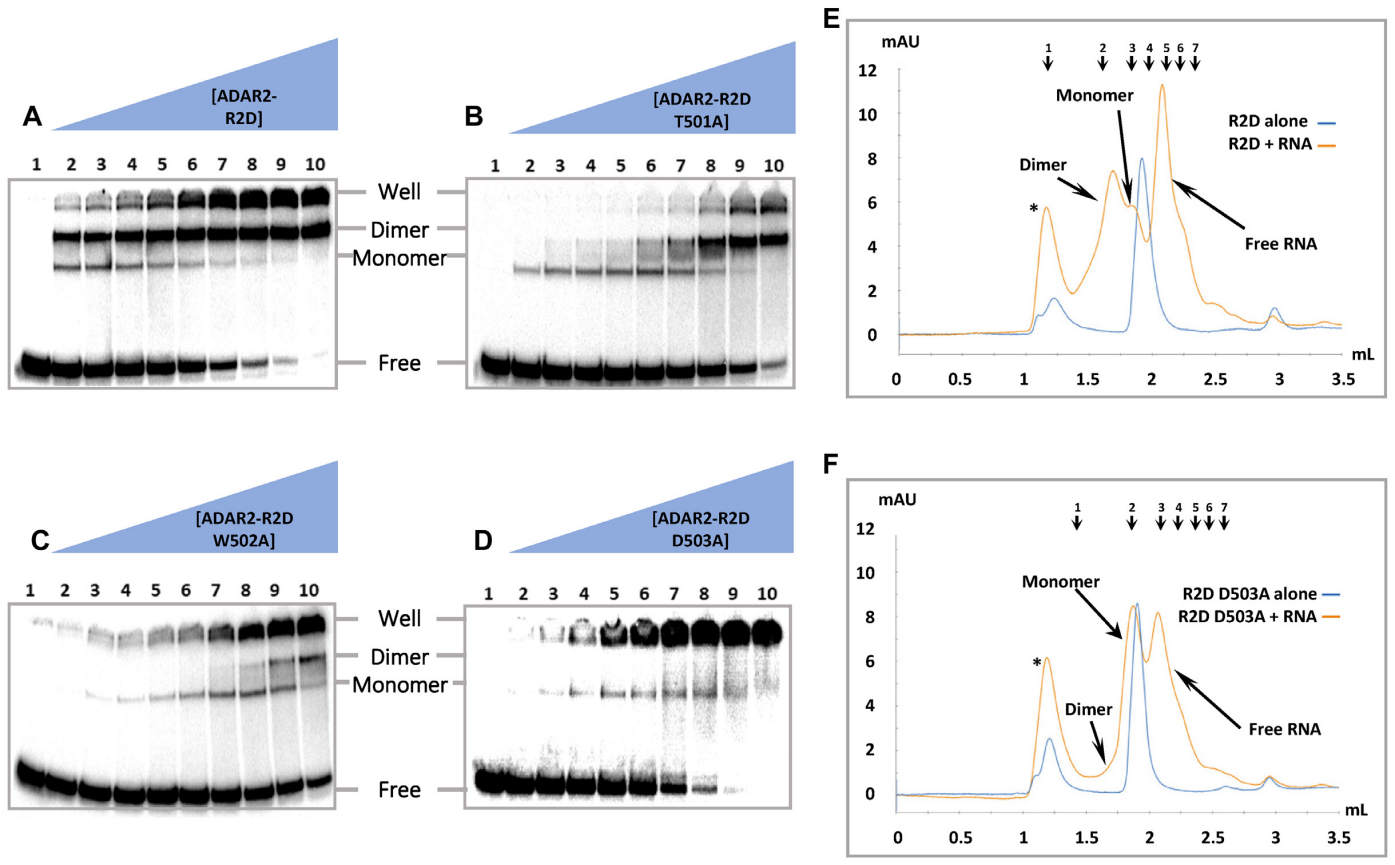
Representative gel shift and gel filtration of hADAR2-R2D E488Q mutants with and without 3′ extended 8-AN RNA. Gel shift and gel filtration experiments were all performed with the E488Q mutation in addition to any other point mutations listed. (**A–D**) Protein concentrations are as follows: 1: no protein, 2: 0.25 nM, 3: 0.5 nM, 4: 1 nM, 5: 2 nM, 6: 4 nM, 7: 8 nM, 8: 16 nM, 9: 32 nM, 10: 64 nM. The band of RNA retained within the well arises from protein–RNA aggregation. (**E, F**) UV trace of hADAR2-R2D eluted from a Superdex Increase 5/150 GL. The elution volumes of each peak for the R2D ± 3′ extend 8-AN RNA runs are as follows: (*) 1.17 ml, dimer 1.71 ml, monomer 1.86 ml, R2D alone 1.91 ml. The elution volumes of each peak for the R2D D503A ± 3′ extended 8-AN RNA runs are as follows: (*) 1.19 ml, monomer 1.88 ml, R2D D503A alone 1.91 ml. The elution peaks of molecular weight standards are indicated and numbered 1–7 at top. The sizes and retention volumes are as follows: (1) thyroglobulin (bovine) 670 kDa, 1.19 ml, (2) γ-globulin (bovine) 158 kDa, 1.59 ml, (3) bovine serum albumin 66 kDa, 1.81 ml, (4) ovalbumin (chicken) 44 kDa, 1.95 ml, (5) carbonic anhydrase 29 kDa, 2.12 ml, (6) myoglobin (horse) 17 kDa, 2.21 ml, (7) cytochrome *c* 12.4 kDa, 2.28 ml. The observed molecular weight corresponding to each protein species can be found in Supplementary Table S3. The peaks designated by an asterisk correspond to protein and RNA eluted within the void volume, representing aggregation.

To evaluate the importance of the protein-protein contacts observed at the dimerization interface, we generated mutant ADAR2-R2D proteins and measured their RNA-binding properties using EMSA and size exclusion chromatography (SEC). For this purpose, each of the residues T501, W502 and D503 were individually mutated to alanine. Focus laid strictly upon residues within the dimerization helix since many of the amino acids in monomer B that comprise the protein-protein interface correspond to key residues of the deaminase catalytic pocket of monomer A. Each alanine mutation resulted in changes to the EMSA banding pattern observed with the 3′ extended 8-AN containing RNA compared to the protein with the wild type dimerization helix (Figure 5A–D). For each mutant, the band representing monomeric RNA binding was largely preserved, while the super shift representing the protein dimer required higher protein concentrations. Comparing the protein concentrations necessary to observe the super shifted band, we noted the severity of the effect to follow the trend D503A > W502A > T501A.

To confirm the banding pattern observed by gel shift represents formation of 1:1 and 2:1 protein–RNA complexes, we performed analytical size-exclusion chromatography (SEC) of ADAR2-R2D and observed its change in retention upon incubation with RNA. Gel filtration of ADAR2-R2D or 8-AN-bearing duplex RNA individually, resulted in a major peak with a retention time consistent with monomeric free protein and a smaller peak corresponding to high molecular weight aggregates. However, combining the protein and RNA together resulted in a gel filtration trace with four major peaks with retention times most consistent with free RNA, 1:1 protein–RNA complex (monomer), 2:1 protein-RNA complex (dimer) and an aggregate peak (Figure 5e) (Supplementary Table S3.) Importantly, the peak corresponding to the ADAR dimer complex disappeared in the SEC run with the D503A mutant, similar to the result obtained by EMSA (Figure 5f). Together the EMSA and SEC results indicate that mutations at residues present on the dimerization helix and shown by crystallography to be involved in protein–protein interaction disrupt RNA-induced dimer formation.

**Figure 6.**
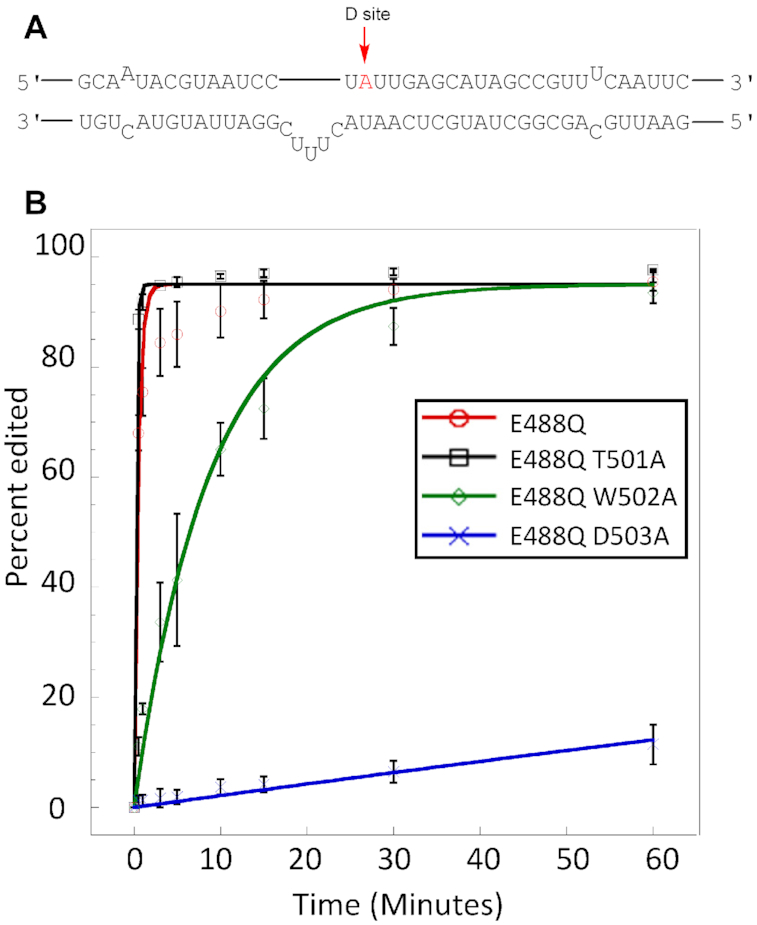
Deamination assays with 100 nM ADAR and 10 nM 5-HT_2c_R D site substrate. (**A**) Local predicted secondary structure of 5-HT_2c_R pre-mRNA used for *in vitro* deamination. (**B**) Deamination kinetics of ADAR2-RD E488Q and dimerization mutants on 5-HT_2c_R substrate (D-site). Conditions for *in vitro* deaminations are stated in Table 2. Error bars represent standard deviation (*n* = 3 technical replicates).

Mutation in the dimerization helix also had a strong effect on *in vitro* editing of the 5-HT2cR substrate (Figure 6). The RNA transcript coding for the human 5-HT2cR contains multiple editing sites and is a well-studied substrate of ADAR1 and ADAR2 (15,56–61). In normal human cells, the D site of this RNA transcript is edited nearly to completion by ADAR2 (56). Furthermore, Eggington and Bass reported that D site editing is inefficient *in vitro* with the ADAR2 deaminase domain alone compared to full length ADAR2, indicating a requirement for dsRBD binding (31). To assess the effect of dimerization helix mutation on RNA editing, we initially carried out *in vitro* editing assays using a transcribed 332 nt RNA fragment of the 5-HT2cR mRNA and the ADAR2-R2D E488Q protein along with the corresponding T501A, W502A and D503A mutants (Figure 6). Importantly, we find that mutation of the dimerization helix affects 5-HT2cR D site editing to a varying degree dependent on the mutant tested. For instance, the D503A mutation reduced the observed rate constant for D site editing by approximately 1000-fold compared to the protein bearing the wild type dimerization helix. The W502A mutation reduced the rate by 17-fold, whereas the T501A mutation was found to be slightly stimulatory despite the loss of a hydrogen bonding interaction with the backbone of residue 488 of the non-catalytic monomer (Table 2). Indeed, the trend observed for changes in rate for *in vitro* editing of the 5-HT2c-R D site follows the same trend seen for disruption of dimer formation described above (D503A>W502A>T501A). For comparison, we also measured rate constants for editing of a 147 nt RNA duplex resembling the GLI1 site, a substrate where dsRBD binding has been shown to be less important for editing efficiency (Supplementary Figure S5) (30). For this substrate, the D503A mutation is not inhibitory and instead has a slight stimulatory effect under the conditions of the *in vitro* assay used (Supplementary Figure S6).

**Table 2.** Single-turnover deamination kinetics of ADAR2-R2D E488Q and dimerization mutants on D-site of the 5-HT_2c_-R substrate^a^.

Enzyme^b^	*k*_obs_ (min^−1^)^c^	*k*_rel_^d^
ADAR2-R2D E488Q	2.13 ± 0.30	1
ADAR2-R2D E488Q, T501A	>3	-
ADAR2-R2D E488Q, W502A	0.12 ± 0.03	0.056
ADAR2-R2D E488Q, D503A	2.32 × 10^−3^ ± 0.76 × 10^−3^	0.001

^a^5-HT_2c_ pre-mRNA substrate sequence as shown in Supplementary Table S1.

^b^ADAR2-R2D reactions were carried out with 100 nM enzyme, 10 nM RNA substrate in 17 mM Tris pH 7.4, 60 mM KCl, 15.6 mM NaCl, 1.6 mM EDTA, 0.003% Nonidet *P*-40, 0.5 mM DTT, 1.0 μg/ml yeast tRNA (Torula), 160 units/ml RNasin.

^c^ *k*_obs_ was calculated by fitting the time course to the equation: [*P*]_*t*_ = α[1 –e^−*k*obs*t*^] where [*P*]_*t*_ is percent edited, α is the end point fitted to 95%, and *k*_obs_ is the observed rate constant.

^d^ *k*_rel_ = *k*_obs_ for mutant/*k*_obs_ for ADAR2-R2D E488Q.

**Figure 7.**
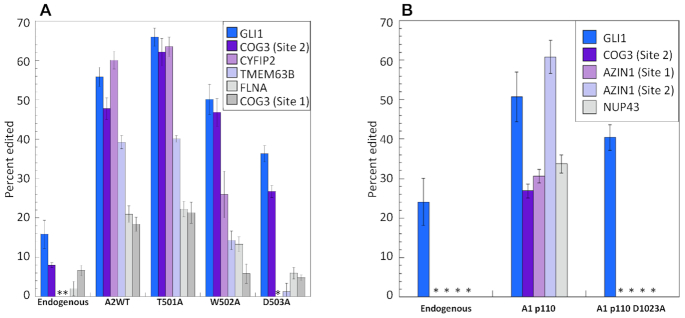
Editing levels of endogenous substrates in HEK293T cells with or without overexpression of ADARs. (**A**) Editing by full length human ADAR2 wild type (A2WT) and dimerization mutants and (**B**) by human ADAR1 p110 wild type (A1 p110) and A1 p110 D1023A mutant. Cells were transfected with 750 ng pcDNA 3.1 containing A2WT or A1 p110 gene and lysed after 48 h. Endogenous editing was measured from cells transfected with empty vector. Asterisks indicate no detected editing. Error bars represent standard deviation (*n* = 3 biological replicates).

To evaluate the effect of the dimerization mutants on the activity of full length ADAR2 in the context of human cells, we overexpressed these proteins in HEK293T cells and quantified editing at known ADAR2 sites (Figure 7A) (62). The effect of these mutations in this experimental context showed a similar trend to that observed in the *in vitro* editing experiments using the ADAR2 R2D mutants. Once again, D503A exhibited the greatest perturbation on editing followed by W502A and T501A (Figure 7A). We also note that the magnitude of the effect varied depending on the substrate analyzed with the GLI1 and COG3 (site 2) affected much less by mutation in the dimerization helix than other targets, such as the CYFIP2 and TEMEM63B transcripts. Western blot analysis of proteins in whole cell lysate was performed for each of the ADAR transfections and confirmed similar expression levels for these ADAR mutants (Supplementary Figure S7a).

Since there is high sequence conservation for residues in the dimerization helix for the different ADAR family members, we also considered the effect of mutation in this location for ADAR1 editing. For this purpose, we generated the human ADAR1 p110 D1023A mutant corresponding to the D503A mutation in human ADAR2 and expressed this protein in HEK293T cells. We measured editing for five known ADAR1 sites in these cells (Figure 7B) (62,63). We found that four of the five sites tested showed a dramatic reduction in editing by ADAR1 as a result of mutation in its dimerization helix (Figure 7B). Again, editing of the GLI1 transcript by overexpressed ADAR1 p110 was found to be only minimally affected by the D1023A mutation. Western blot analysis of proteins in lysate from cells transfected with ADAR1 p110 confirmed a similar expression level for the D1023A mutant compared to WT protein (Supplementary Figure S7b).

## DISCUSSION

Several earlier studies provided biochemical evidence that the ADAR proteins can dimerize (32–38). O’Connell and colleagues reported that *Drosophila* ADAR is capable of dimerization in an RNA-dependent manner (33). They identified the minimal region required for dimerization to consist of the N-terminus and dsRBD1, a sequence absent in the constructs of human ADAR2 used in the structural studies described here. Furthermore, FRET analysis of fluorescent fusion proteins containing human ADAR1 and ADAR2 by MacMillan *et al.* also supported ADAR dimerization (35). It should be noted that the FRET results supported the hypothesis that dimerization is mediated by the N-terminal regions of the protein. However, this conclusion was reached by showing a lack of FRET with the deaminase domain of ADAR2 alone and their study did not include a construct containing dsRBD2 (35). Nishikura and colleagues showed that mutants of ADAR1 or ADAR2 missing key dsRBD residues essential for RNA binding can still dimerize as indicated by a co-immunoprecipitation assay (32,36). They concluded ADAR2 is capable of homodimerization and ADAR1 can homodimerize, as well as complex with Dicer, in an RNA independent manner. They also observed ADAR1 p110 dimerization when the deaminase domain of one monomer was removed (32,36). Thus, these previous reports in the literature indicated that ADARs can form homodimers or heterodimers and that dimerization can be RNA-dependent or RNA-independent (33–39). Additionally, some of these studies have also suggested that dimerization is required for ADAR catalytic activity as is the case for the tRNA-modifying ADAT enzymes (64).

In none of the previous studies on ADAR dimerization has a dimerization interface been structurally characterized and the importance of the observed protein-protein contacts been defined. In this work, we describe in structural detail an ADAR dimer interface involving a surface helix whose sequence is highly conserved in the ADAR family and the binding pocket for this helix across the dimer interface that has two functions; in one monomer it serves as the RNA substrate recognition site and in the other monomer it is involved in protein-protein interactions. Mutagenesis of key residues displayed on the helix disrupts dimerization and, for most RNA substrates tested, is highly detrimental to the editing reaction. Interestingly, the ADAR dimerization described here involves an interface that includes both protein-protein and protein-RNA contacts. Indeed, ADAR dimerization through contacts between deaminase domains in each monomer appears to be enhanced by dsRBD2 binding to the adjacent RNA duplex (Supplementary Figure S4, Table S4). However, RNA binding is not absolutely required to form the protein-protein contacts observed here. It is apparent that high protein concentration can also lead to this deaminase–deaminase interaction since this interface was also observed in the crystal packing of the RNA-free ADAR2d crystal structure reported previously and in crystals of ADAR2d bound to RNA in a 2:1 ratio (28,29). Under the conditions of our analytical gel filtration, the ADAR2-R2D construct behaves as a monomer in the absence of RNA, suggesting the protein-protein interaction alone is insufficient for stable dimer formation at that protein concentration without the assistance of RNA binding. It is possible that domains missing in this construct also contribute to dimerization of full length ADAR2, as some studies suggest dsRBD1 has a larger role in ADAR2 dimerization than dsRBD2 (37).

The crystal structure reported here also reveals an important role for ADAR dimerization in RNA substrate recognition. We find that dimerization of ADAR2 deaminase domains allows one domain to participate directly in catalysis of adenosine deamination while the other positions its covalently linked dsRBD for contact to the RNA duplex on the 3′ side of the editing site (Figure 4A). Therefore, dimer formation facilitates simultaneous contact of a deaminase domain and a dsRBD on the same RNA molecule. Interestingly, we find that disrupting the dimer by mutagenesis inhibits RNA editing, but in an RNA substrate-specific manner. For instance, editing of the 5-HT_2c_R D site is highly dependent on an intact dimer interface as blocking dimer formation substantially inhibits editing on this substrate. On the other hand, dimerization appears to be less important for editing the GLI1 RNA substrate. It is important to note that, for the GLI1 substrate used here, the editing-site 5′ and 3′ nearest neighbor nucleotides, the identity of the orphan base and the position of the editing site on the RNA duplex are nearly ideal for reaction with the ADAR2 deaminase domain (28). It is likely that, for such a substrate, dimerization to position a dsRBD for additional contact to the RNA is unnecessary for efficient editing. It remains to be seen what fraction of endogenous ADAR substrates are sensitive to inhibition of dimerization. For the six ADAR2 sites analyzed here from HEK293 cells, four (CYFIP2, TMEM63B, FLNA and COG3 (site 1)) showed a substantial decrease in editing with the ADAR2 dimer interface mutants whereas two sites (GLI1 and COG3(site 2)) were less dependent. In addition, four of the five ADAR1 sites tested here showed a large decrease in editing resulting from mutation in its dimerization helix with only the GLI1 substrate unaffected (Figure 7). Given the apparent significance of ADAR dimerization in editing, it will be important to identify molecules capable of inhibiting dimerization and test their effect in ADAR-dependent pathways. For instance, it is possible that ADAR1 dimerization inhibitors will be particularly lethal to cancer cells that show an interferon-stimulated gene signature (65,66). The structural data presented here should enable structure-guided approaches to the identification of ADAR dimerization inhibitors.

Recent studies on the catalytically inactive isoform ADAR3 has revealed knockout of exon 3 of the *Adarb2* gene, which encodes its two double stranded RNA binding domains, results in altered behavior related to anxiety and learning in mice (67). Additionally, titration of recombinant ADAR3 has shown to have in inhibitory effect on the activity of ADAR1 and ADAR2 in vitro (68). ADAR3 shares the conserved dimerization helix, which may permit heterodimerization. Despite being catalytically inactive, it is possible to explain ADAR3′s effect on behavior in mice and in vitro activity to be facilitated by the dimerization helix, although further experiments must be conducted to confirm this.

The ADAR2 dimer described here involves one ‘catalytic’ monomer and one monomer that appears to be used mainly for RNA substrate recognition. A similar division of labor between homologous subunits has been observed in other DNA and RNA modifying enzymes (69–72). Efficient generation of m^6^A by the methyltransferase Mettl3 requires heterodimerization with structurally similar Mettl14 (69,70). Analogous to dimerization in ADAR, the subunit Mettl3 is primarily responsible for methyltransferase activity, while Mettl14 forms a stabilizing junction through an anti-parallel interaction of the two enzymes’ α2 helices and β4 strands (69,70). Mutational analysis of each subunit shows loss of activity upon disruption of Mettl3′s putative active site, but methylation activity is insensitive to the analogous mutations in Mettl14 (69). While there currently are no structures of Mettl3–Mettl14 complexed with an RNA strand, heterodimerization unveils the proposed RNA binding surface (69,70). Indeed, alanine or charge-reversal mutations of the positively charged residues on either monomer of the proposed RNA binding groove results in significant decreases to methylation activity (69–71). It is possible for the Mettl3–Mettl14 complex to behave similarly to ADAR by which heterodimerization results in formation of an RNA binding surface that gives rise to the enzymes’ sequence specificity. Another pair of RNA modifying enzymes that act as an asymmetric dimer are the human tRNA methyltransferases Trm6 and Trm61 (72). Trm61 acts as the catalytic unit, while the non-catalytic Trm6 serves primarily as an RNA binding scaffold (72). Unlike the asymmetric dimer observed in the ADAR and Mettl3/14 crystal structures Trm6 and Trm61 forms a dimer of dimers, but the tetrameric complex maintains a similar protein:RNA ratio of 4:2 where each tRNA is bound primarily by a single Trm6/61 pair in the complex (72).

While the structure and follow up experiments reported here significantly advance our understanding of ADAR dimerization and RNA substrate recognition, important questions remain. The ADAR2 fragment crystallized in complex with RNA in this study lacks the first 214 amino acids from the N-terminus of the full-length enzyme, including dsRBD1. Thus, how ADAR2 dsRBD1 coordinates with dsRBD2 and the deaminase domain remains an open question. In addition, while our footprinting experiment identified an important dsRBD binding site 3′ to the editing site, we also noted protection of the duplex over two helical turns (>24 bp) 5′ to the editing site. It is possible that this protection arises from RNA contact with the dsRBD from monomer A not resolved in our structure. However, additional crystallization or cryoEM studies with longer RNAs will be necessary to test this hypothesis. Finally, no high-resolution structural data has been reported for ADAR1 in complex with RNA. Such data are needed to fully understand differences in RNA substrate selectivity for the different members of the ADAR family.

## DATA AVAILABILITY

Atomic coordinates and structure factors have been deposited in the Protein Data bank under the accession code 6VFF.

## Supplementary Material

gkaa532_Supplemental_File
