# Supplementary material for: Asymmetric dimerization of adenosine deaminase acting on RNA facilitates substrate recognition
Source: Nucleic Acids Res. 2020 Jun 29;48(14):7958–72. doi: 10.1093/nar/gkaa532 (PMC7641318; doi:10.1093/nar/gkaa532)
Supplement: gkaa532_Supplemental_File [file gkaa532_supplemental_file.pdf]

Supporting information

Asymmetric Dimerization of Adenosine Deaminase acting on RNA Facilitates Substrate Recognition

Alexander S. Thuy-Boun[1], Justin M. Thomas[1], Herra L. Grajo[1], Cody M. Palumbo[1], SeHee Park[1], Luan T. Nguyen[1], Andrew J. Fisher[1,2] and Peter A. Beal[1]

[1]Department of Chemistry, University of California, Davis, CA, USA.

[2]Department of Molecular and Cellular Biology, University of California, Davis, CA, USA.

Correspondence to: pabeal@ucdavis.edu, ajfisher@ucdavis.edu

Contents:

Figure S1. Linear plot of optical density of MPE-Fe cleavage fragments.....S3

Figure S2. Calibration curve of molecular weight standards for gel filtration chromatography.....S3

Figure S3. Sequence of the in-vitro transcribed $5HT_{2C}$ RNA used in in-vitro deamination.....S4

Figure S4. Gel shift assay of A2-R2D and hGLI1 RNA duplexes.....S5

Figure S5. Sequence of the in-vitro transcribed hGLI1 RNA used for in-vitro deamination.....S6

Figure S6. In-vitro deamination of A2-R2D and in-vitro transcribed hGLI1 RNA.....S7

Figure S7. Western blot of HEK293T cell lysate expressing A2FL or A1 p110.....S7

Table S1. DNA Primer sequences……………………………………………………………………………………………S8-S9

Table S2. Sequence of oligonucleotides used for gel shift assays and x-ray crystallography…………………….S9

Table S3. Molecular weight of protein-RNA complexes by gel filtration chromatography………………………S9

Table S4. Fitted microscopic dissociation constant and Hill coefficient by gel shift assay……………………….S10

Supplementary methods………………………………………………………………………………………………….S10-11

References……...S11

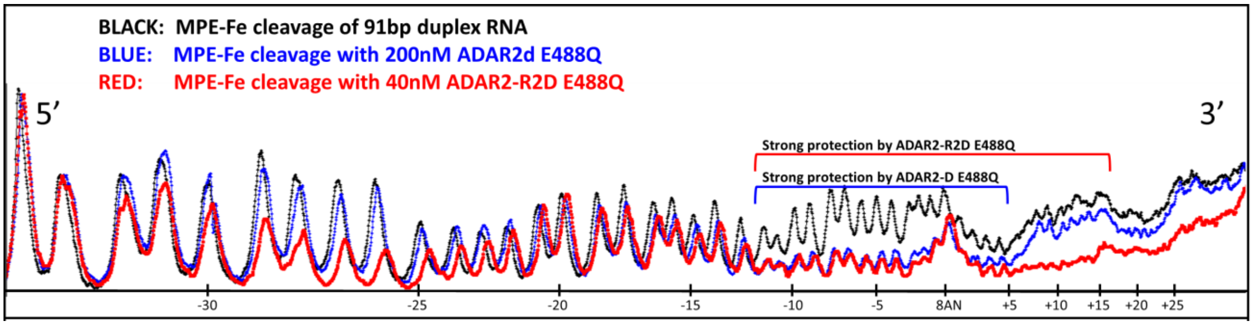

Fig. S1. Linear plot of optical density of MPE-Fe cleavage fragments. X-axis shows position of cleavage fragments relative to 8-AN. Black: No protein, Blue: 200 nM hADAR2-d, 40nM hADAR2-R2D.

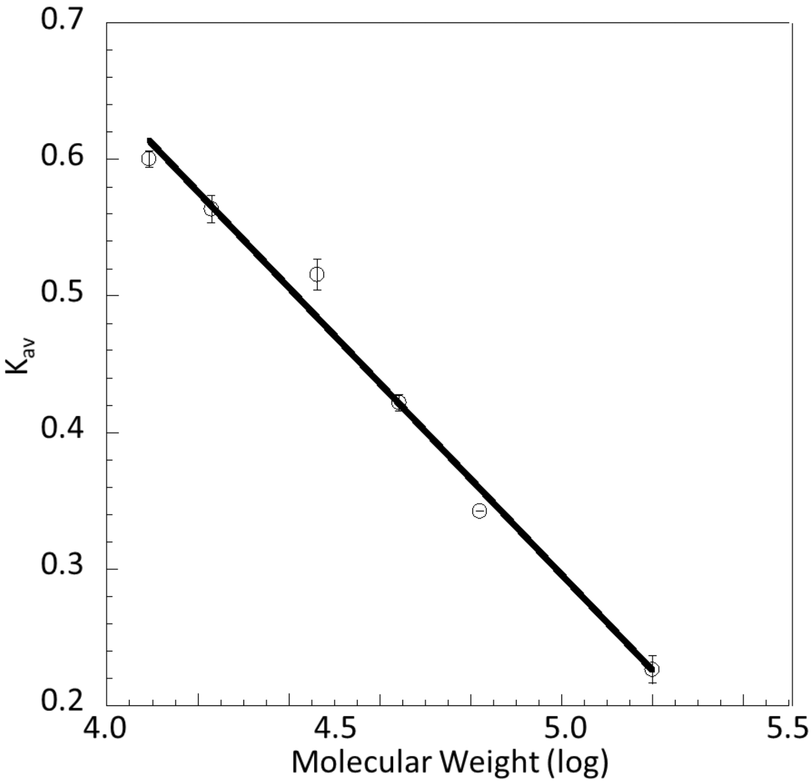

Fig. S2. Calibration curve used to estimate molecular weight of ADAR + RNA complexes. Gel filtration chromatography column Superdex 200 5/150 GL was calibrated using thyroglobulin (670 kDa), γ-globulin (158 kDa), bovine serum albumin (66 kDa), ovalbumin (44 kDa), carbonic anhydrase (29 kDa), myoglobin (17 kDa), cytochrome C (12 kDa). The calibration curve was plotted using the gel-phase distribution coefficient (K_{av}) versus the logarithm of the molecular weight (Log MW). $K_{av} = (V_e-V_o)/(V_c-V_o)$ where V_e = elution volume, V_o = column void volume (1.19 mL based on thyroglobulin elution volume), V_c = geometric column volume (3 mL). Straight line is the calibration curve calculated from the data for molecular weight standards ($R^2 = 0.9929$). The equation Y = -0.35081*X + 2.0499 was used to calculate the experimental molecular weights reported in **Table S3.** Error bars represent standard deviation (n = 3 technical replicates).

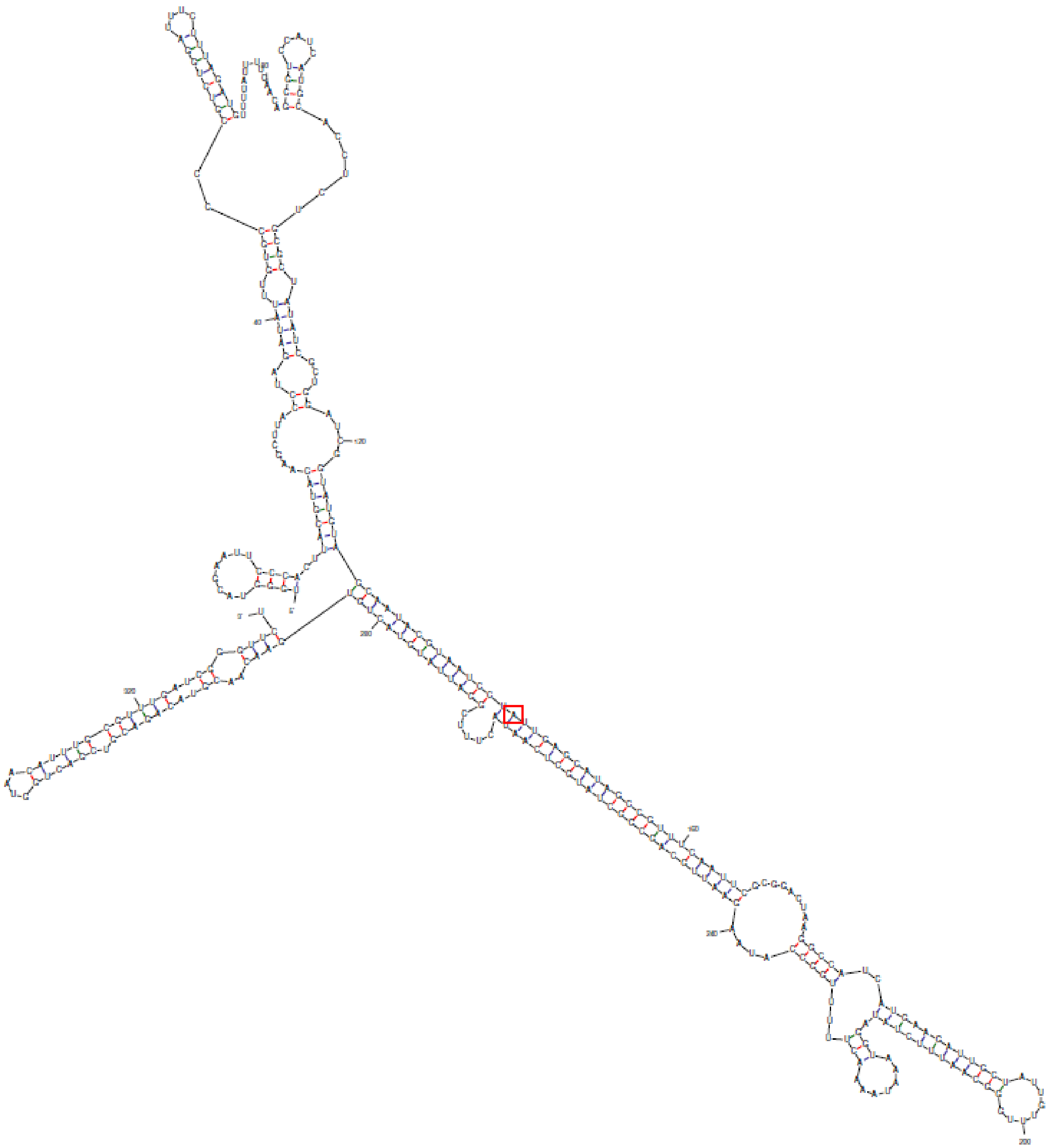

5′-UGGGUACGAAUUCCCACUUACGUACAAGCUUACCUAGAUAUUUGUGCCCCGUCUGGAUUUCUUUAGA
UGUUUUAUUUUCAACAGCGUCCAUCAUGCACCUCUGCGCUAUAUCGCUGGAUCGGUAUGUAGCAAUACGU
AAUCCUAUUGAGCAUAGCCGUUUCAAUUCGCGGACUAAGGCCAUCAUGAAGAUUGCUAUUGUUUGGGCAA
UUUCUAUAGGUAAAUAAAACUUUUUGGCCAUAAGAAUUGCAGCGGCUAUGCUCAAUACUUUCGGAUUAUG
UACUGUGAACAACGUACAGACGUCGACUGGUAACAUUUGCGUUUGAUCGGGUUCU-3′

Fig. S3. 2D structure predicted by mfold (1) and sequence of the in-vitro transcribed 332mer 5HT$_{2C}$ RNA used in in-vitro deamination. Deaminated D-site adenosine is shown in red.

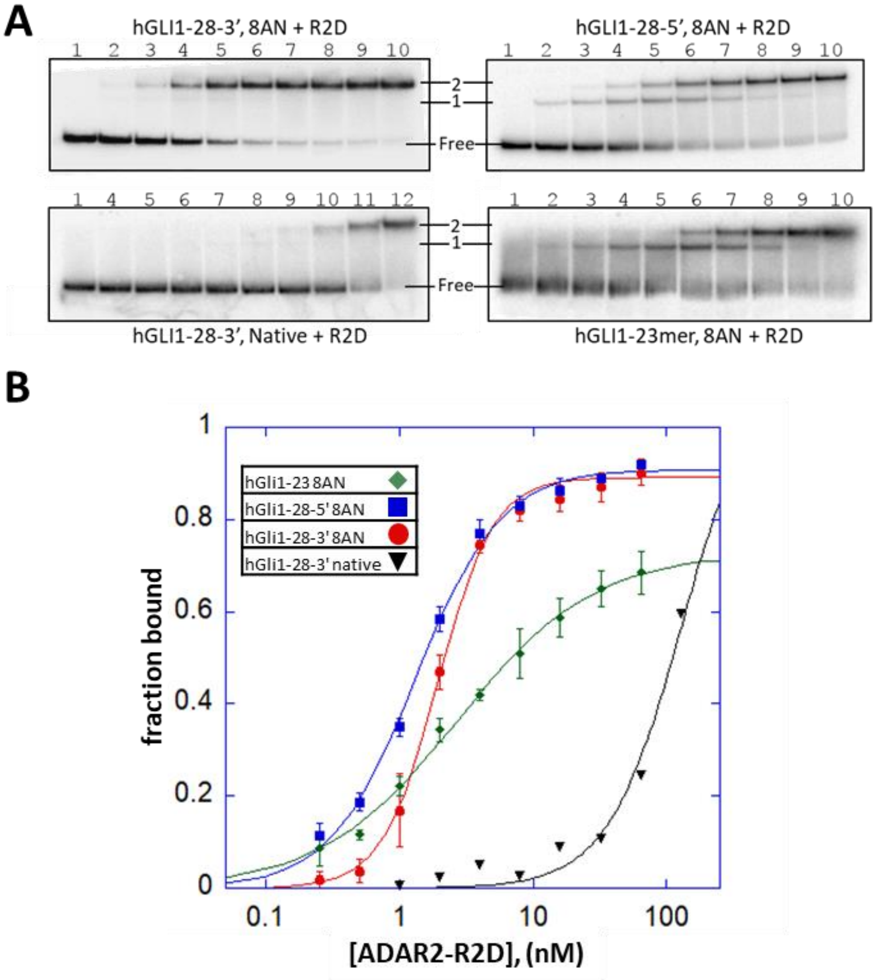

Fig. S4. Electrophoretic mobility gel shift assay of ADAR2-R2D and hGli1 RNA duplexes. (A) Representative gel shifts of ADAR2-R2D and hGLI1 RNA duplexes. Lane 1: no protein added, Lanes 2-12: 0.25, 0.5, 1, 2, 4, 8, 16, 32, 64, 128 and 256 nM ADAR2-R2D. (B) Fitted plot of RNA fraction bound vs concentration of ADAR2-R2D. Data was plotted using KaleidaGraph and the line of best fit was calculated using the Hill Equation (m2/(1+(m1/x)^m3)) where x = [ADAR2-R2D], m2 = binding endpoint, m1 = K_A (microscopic dissociation constant), m3 = Hill coefficient. Error bars represent standard deviation (n = 3 biological replicates). Fitted K_A and Hill coefficient can be found in **Table S4**.

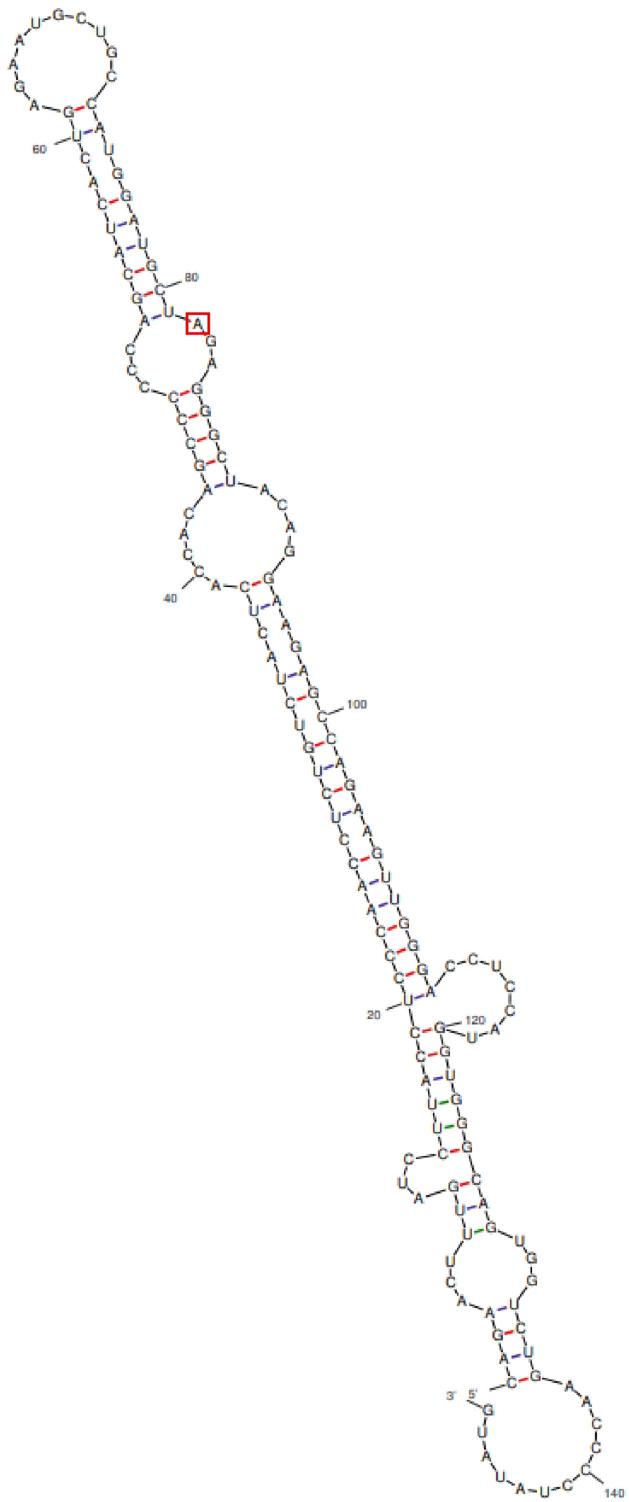

5′-CAGAACUUUGAUCCUUACCUCCCAACCUCUGUCUACUCACCACAGCCCCCCAGCAUCACUGAGAAUG
CUGCCAUGGAUGCUAGAGGGCUACAGGAAGAGCCAGAAGUUGGGACCUCCAUGGUGGGCAGUGGUCUGAA
CCCCUAUAUG-3′

Fig. S5. 2D structure predicted by mfold (1) and sequence of the in-vitro transcribed hGLI1 147mer RNA used for in-vitro deamination. Deaminated adenosine is shown in red.

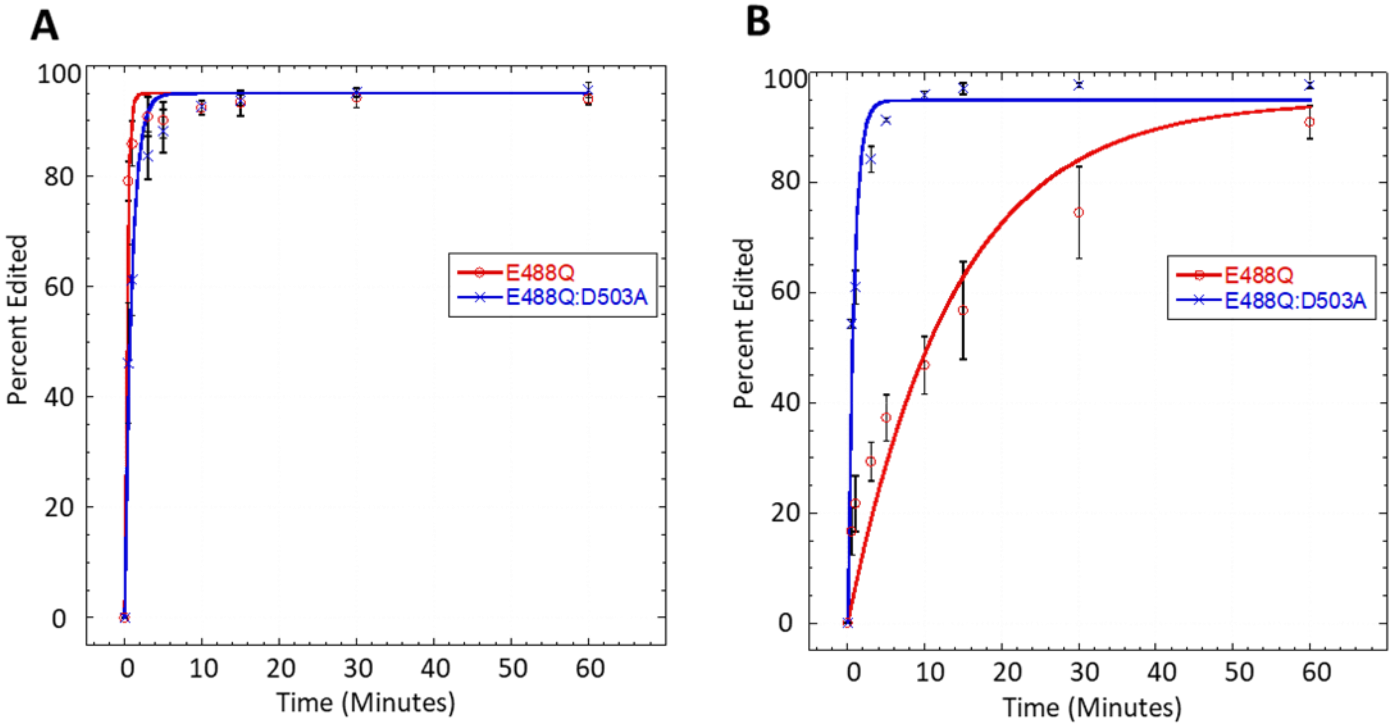

Fig. S6. In-vitro deamination assays with ADAR and hGLI1 147mer substrate. (a) Deamination kinetics of 100 nM ADAR2-R2D E488Q and 10 nM RNA. (b) Deamination kinetics of 10 nM ADAR2-R2D E488Q and 10 nM RNA. Reactions were carried out in 17 mM Tris pH 7.4, 60 mM KCl, 16 mM NaCl, 2 mM EDTA, 0.003% Nonidet P-40, 0.5 mM DTT, 1.0 µg/mL yeast tRNA (Torula), 160 units/mL RNasin.

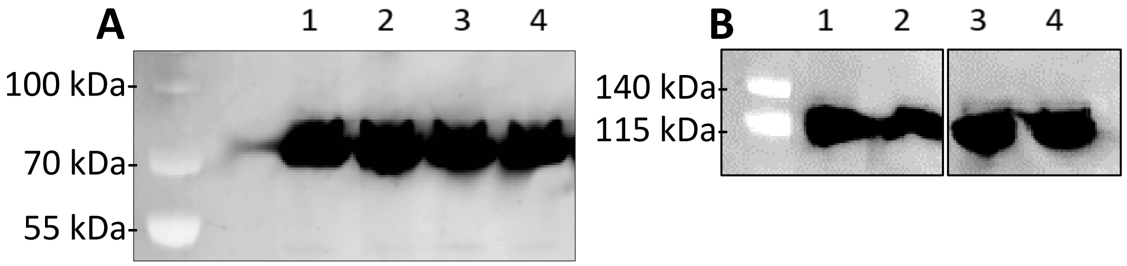

Fig. S7. Western blot analysis of whole cell lysate from HEK293T cells expressing full length ADAR WT and dimerization mutants. (A) Western blot of HEK293T cells transfected with 750 ng pcDNA 3.1 containing ADAR2 gene and lysed after 48 h. Protein construct of each lane are as follows: 1. ADAR2 FLWT, 2. ADAR2 FL T501A, 3. ADAR2 FL W502A, 4. ADAR2 FL D503A. Expected MW of full length ADAR2 is approximately 75 kDa. (B) Western blot of HEK293T cells transfected with 500 ng (Lanes 1 & 3) or 750 ng (Lanes 2 & 4) pcDNA 3.1 containing ADAR2 gene and lysed after 48 h. Protein construct of each lane are as follows: 1 & 2. ADAR1 p110 WT, 3 & 4. ADAR1 p110 D503A. Expected MW of ADAR1 p110 is approximately 110 kDa. The western blot is cropped into two images as the lanes are apart spatially within the same membrane.

Table S1. DNA Primer sequences

Primer Sequences for site directed mutagenesis

E488Q FWD	5′-GACCAAAATAGAGTCTGGTCAGGGGACGATTCCAGTGCGCTC-3′
E488Q RVS	5′-GAGCGCACTGGAATCGTCCCCTGACCAGACTCTATTTTGGTC-3′
T501A FWD	5′-AATGCGAGCATCCAAGCTTGGGACGGGGTGCTG-3′
T501A RVS	5′-CAGCACCCCGTCCCAAGCTTGGATGCTCGCATT-3′
W502A FWD	5′-GCGAGCATCCAAACGGCTGACGGGGTGCTGCAA-3′
W502A RVS	5′-TTGCAGCACCCCGTCAGCCGTTTGGATGCTCGC-3′
D503A FWD	5′-GAGCATCCAAACGTGGGCTGGGGTGCTGCAAGGGG-3′
D503A RVS	5′-CCCCTTGCAGCACCCCAGCCCACGTTTGGATGCTC-3′

Forward primer sequences to PCR amplify T7 promoter and substrate and sequence RT-PCR product of in-vitro deamination.

hGLI1 +T7 FWD	5′-TAATACGACTCACTATAGGGAATATTAAGCTTGG-3′
hGLI1 FWD	5′-CAGAACTTTGATCCTTACCTC-3′
hGLI1 RVS	5′-GCAGTGGTCTGAACCCCTATATG-3′
5HT2c-R + T7 FWD	5′-TAATACGACTCACTATAGGGCGAATTGG-3′
5HT2c-R FWD	5′-TGGGTACGAATTCCCACTTACGTACAAGCTT-3′
5HT2c-R RVS	5′-GTAACATTTGCGTTTGATCGGGTTCT-3′

Primer sequences for RT-PCR and nested RT-PCR to quantify editing of endogenous sites in HEK293T cells.

TMEM63B RT FWD	5′-CCGCTGGCTCTTTGATAAGAAATTCTTGGCTGAGG-3′
TMEM63B RT RVS	5′-AGCCAGAAGAGGCAGAGGATGGGCG-3′
TMEM63B Nest FWD	5′-CAGCTATTCGGTTTGAGTGTGTGTTCC-3′
TMEM63B Nest RVS	5′-CGGCCACCACCTGGTTCACAGCCC-3′
CYFIP2 RT FWD	5′-TCCTGGCCAACCACAACAGGATCACCCAGTGTC-3′
CYFIP2 RT RVS	5′-TAGGTCGAAGAGCTCGCGATACTCCTCGTCTG-3′
CYFIP2 Nest FWD	5′-TCCACCAGCAACTTGAAGTGATCCCAGGCTATGA-3′
CYFIP2 Nest RVS	5′-ACTTCTGGCTGTCCAGCCCTGAGCCCG-3′
FLNA RT FWD	5′-TCAGTATCTGGACCCGGGAAGCTGGTGC-3′
FLNA RT RVS	5′-TGCCGTTGAACTTGACGTCAATCAGGTAAACGCC-3′
FLNA Nest FWD	5′-TGGAGGCCTGGCCATTGCTGTCGAGGG-3′
FLNA Nest RVS	5′-ATTCTCCCGAGGGATGAAGCGCACAGC-3′
AZIN1 RT FWD	5′-GAAGGATCTGGTGTTAAGATAATTTCAGAACCCG-3′
AZIN1 RT RVS	5′-ACTGGAATGTTGACCAGACAAGCTTAACC-3′
AZIN1 Nest FWD	5′-GAAGCTACTATGTGTCTTCTGCATTTACAC-3′
AZIN1 Nest RVS	5′-TGCAACTTCAGATCTAAAGAAGCGT-3′
COG3 RT FWD	5′-CAGATGCATAGATAGGGCAGTGTTCCAAGGA-3′
COG3 RT RVS	5′-ACCTTTGTCATGAACTCCTCCAGCTGTTC-3′
COG3 Nest FWD	5′-TTATCACAGGAAGCATTGTCTGCCTGCATTCAGTC-3′
COG3 Nest RVS	5′-TACAAACAGCTTGGTCTGCTGCTGAAT-3′
NUP43 RT FWD	5′-TCTTTCCAAACTTTGTTAGATTTTAAATGTATTATTGACCTGAGA-3′
NUP43 RT RVS	5′-CGGTGTTACATTACATAAAGCTTAGTTTCTTATTAAAATTGGCAA-3′
NUP43 Nest FWD	5′-CCTTAATGACAAATCACTGCTATTAGACAATTG-3′

NUP43 Nest RVS	5′-AACTTGTCAATTGGCATGAAATGTAATGCT-3′
GLI1 RT FWD	5′-CGAGCCGAGTATCCAGGATACAAC-3′
GLI1 RT RVS	5′-CCCATATCCCAGAGTATCAGTAGGTGG-3′
GLI1 Nest FWD	5′-CCCAATGCAGGGGTCACCCGGAGGG-3′
GLI1 Nest RVS	5′-GAAGTCCATATAGGGGTTCAGACCACTGCCCAC-3′

Table S2. Sequence of oligonucleotides used for gel shift assays, foot printing and x-ray crystallography (N=8-azanebularine)

Sequences for gel shift assay

hGLI1 3′ Extend Top	5′-GCUCGCGAUGCUNGAGGGCUCUGAUAGC-3′
hGLI1 3′ Extend Bot	5′-GCUAUCAGAGCCCCCCAGCAUCGCGAGC-3′
hGLI1 5′ Extend Top	5′-GCAUAGCUCGCGAUGCUNGAGGGCUCUG-3′
hGLI1 5′ Extend Bot	5′-CGUAUCGAGCGCUACGACCCCCCGAGAC-3′
hGLI1 23mer Top	5′-GCUCGCGAUGCUNGAGGGCUCUG-3′
hGLI1 23mer Bot	5′-CGAGCGCUACGACCCCCCGAGAC-3′

Sequences for MPE-Fe foot printing

hGLI1 91mer 5′ end	5′-GAUCGAUCCAGAUGCAGCAAGUCCACGUGCAUG-3′
hGLI1 91mer 3′ end	5′-GGAUGGACAUCGACGAUCUGGACGUGCAAG-3′
GLI1 DNA splint	5′-TTGCACGTCCAGATCGTCGATGTCCATCCGCTATCAGAGCCCTC TAGCATCGCGAGCCATGCACGTGGACTTGCTGCATCTGGATCGATC-3′
hGLI1 91mer bottom	5′-CUUGCACGUCCAGAUCGUCGAUGUCCAUCCGCUAUCAGAGCCCU CUAGCAUCGCGAGCCAUGCACGUGGACUUGCUGCAUCUGGAUCGAUC-3′

Sequences for crystallography

hGLI1 32mer Top	5′-GCUCGCGAUGCUNGAGGGCUCUGAUAGCUACG-3′
hGLI1 32mer Bot	5′-CGUAGCUAUCAGAGCCCCCCAGCAUCGCGAGC-3′

Table S3. Observed molecular weight of protein-RNA complexes by gel filtration chromatography.

R2D E488Q + RNA	Observed molecular weight[a] (kDa)	Calculated molecular weight (kDa)
Free Protein	51 ± 1	55
Monomer	62 ± 3	74
Dimer	107 ± 5	130

R2D E488Q D503A + RNA	Observed molecular weight (kDa)	Calculated molecular weight (kDa)
Free Protein	51 ± 1	55
Monomer	58 ± 1	74
Dimer	-	130

[a] Observed molecular weights were calculated by fitting average K_{av} to the equation Y = -0.35081*X + 2.0499 derived from figure **S7.** The values reported include the standard deviation (n = 3 biological replicates).

Table S4. Fitted microscopic dissociation constant (K_A) and Fitted Hill coefficient of hGli1 RNA duplexes and ADAR2-R2D. The values reported include the standard deviation (n = 3 biological replicates).

RNA	Fitted microscopic dissociation constant (K_A)	Fitted Hill coefficient
hGLI1-23 8AN	2.67 ±0.38nM	0.85
hGLI1-28-5' 8AN	1.31 ±0.07nM	1.37
hGLI1-28-3' 8AN	1.79±0.13nM	2.12
hGLI1-28-3' native	>100nM	N/A

Supplementary methods

Preparation of in vitro transcribed hGLI1-RNA for in vitro deamination kinetics. The GLI1 transcript was prepared as described previously (2). In brief, a truncation including 81 nt upstream and 65 nt downstream of the editing site was inserted into a pYES3/CT yeast expression vector and linearized with XhoI. The RNA was transcribed from linearized plasmid using the ThermoFisher Megascript T7 kit (AM1334). RNA generated from the transcription reaction was purified as described above. See **Fig. S5.** For transcribed RNA sequence.

Deamination kinetics with ADAR2-R2D and hGLI1-RNA. Deamination reactions had a final volume of 10 µL with concentrations of 10 nM RNA and either 100 nM or 10 nM ADAR2-R2D. The final reaction solution contained 17 mM Tris pH 7.4, 60 mM KCl, 16 mM NaCl, 2 mM EDTA, 0.003% Nonidet P-40, 0.5 mM DTT, 1.0 µg/mL yeast tRNA, 160 units/mL RNasin. The reaction was incubated at 30 °C. Time points include 0, 0.5, 1, 3, 5, 10, 15, 30 and 60 min. The zero point consisted of omitting protein, and immediate quench. Reactions were quenched by addition of 190 µL of 95 °C nuclease free H_2O and incubation for 5 min at 95 °C. cDNA was generated from RNA via RT-PCR using Access RT-PCR kit (Promega) for 24 cycles. PCR product was purified using DNA Clean and Concentrator kit (Zymo). Purified samples were subjected to Sanger Sequencing and sequence traces were analyzed by 4Peaks (Nucleobytes) to quantify percent editing. Rate constants were calculated by fitting the time course to the equation: $[P]_t = \alpha[1=e^{-kobst}]$ where $[P]_t$ is percent edited, α is the end point fitted to 95 percent, and k_{obs} is the observed rate constant. See **Table S1.** for cDNA sequencing primers.

Detection of full length hADAR protein in transfected HEK293T cells. HEK293T cells were cultured in Dulbecco's Modified Eagle Medium (DMEM), 10% Fetal Bovine Serum (FBS), and 1% Anti-Anti at 37 °C and 5% CO2. At 70-90% confluency, 8 x 10^4 cells for ADAR2 experiments, or 6.4 x 10^3 cells for ADAR1 experiments were seeded into a 96 well plate. After 24 h, cells were transfected with 750 ng pcDNA3.1 plasmid containing ADAR1 p110 or full length hADAR2 WT or mutant with HA tag. Transfection was carried out using Lipofectamine 2000. After incubation of transfection reagent and plasmid in Opti-MEM, the solution was added to designated well and incubated at 37 °C and 5% CO2 for 48 h. Cells were lysed with 300 µL of lysis buffer for ADAR1 detection, or 50 µL of lysis buffer for ADAR2 detection (50 mM Tris-HCl, pH 8.0, 150 mM NaCl, 1% (v/v) NP-40, supplemented with Halt protease inhibitor cocktail with shaking at 4 °C for 30 min. Samples were resolved on an SDS-PAGE gel alongside Page Ruler Prestained Plus Protein Ladder. Western blotting was carried out using an HA tag monoclonal antibody (2-2.2.14 ThermoFisher) as primary antibody at 1:10,000 dilution and anti-mouse IgG with alkaline phosphatase-conjugated secondary antibody (Santa Cruz Biotechnology) at

1:2,000 dilution. ADAR proteins were detected using an ECF substrate (GE Healthcare) on a Typhoon Trio Variable Mode Imager (GE Healthcare) **Fig. S7.**

Supplementary references

1. Zucker. M. (2003). Mfold Web Server for Nucleic Acid Folding and Hybridization Prediction. Nucleic Acids Res. 31 (13), 3406-3415.
2. Schirle, N.T., Goodman, R.A., Krishnamurthy, M., and Beal, P.A. (2010). Selective inhibition of ADAR2-catalyzed editing of the serotonin 2c receptor pre-mRNA by a helix-threading peptide. Org. Biomol. Chem. *8*, 4898–4904.
